# CRL4-DCAF12 Ubiquitin Ligase Controls MOV10 RNA Helicase during Spermatogenesis and T Cell Activation

**DOI:** 10.3390/ijms22105394

**Published:** 2021-05-20

**Authors:** Tomas Lidak, Nikol Baloghova, Vladimir Korinek, Radislav Sedlacek, Jana Balounova, Petr Kasparek, Lukas Cermak

**Affiliations:** 1 Laboratory of Cancer Biology, Institute of Molecular Genetics of the Czech Academy of Sciences, 252 42 Vestec, Czech Republic; tomas.lidak@img.cas.cz (T.L.); nikol.baloghova@img.cas.cz (N.B.); vladimir.korinek@img.cas.cz (V.K.); 2 Faculty of Science, Charles University, 128 00 Prague, Czech Republic; 3 Laboratory of Cell and Developmental Biology, Institute of Molecular Genetics of the Czech Academy of Sciences, 252 42 Vestec, Czech Republic; 4 Czech Centre for Phenogenomics, Institute of Molecular Genetics of the Czech Academy of Sciences, 252 50 Vestec, Czech Republic; radislav.sedlacek@img.cas.cz (R.S.); jana.balounova@img.cas.cz (J.B.); petr.kasparek@img.cas.cz (P.K.)

**Keywords:** DCAF12, WDR40A, MOV10, C-terminal degron, spermatogenesis, T cell activation

## Abstract

Multisubunit cullin-RING ubiquitin ligase 4 (CRL4)-DCAF12 recognizes the C-terminal degron containing acidic amino acid residues. However, its physiological roles and substrates are largely unknown. Purification of CRL4-DCAF12 complexes revealed a wide range of potential substrates, including MOV10, an “ancient” RNA-induced silencing complex (RISC) complex RNA helicase. We show that DCAF12 controls the MOV10 protein level via its C-terminal motif in a proteasome- and CRL-dependent manner. Next, we generated *Dcaf12* knockout mice and demonstrated that the DCAF12-mediated degradation of MOV10 is conserved in mice and humans. Detailed analysis of Dcaf12-deficient mice revealed that their testes produce fewer mature sperms, phenotype accompanied by elevated MOV10 and imbalance in meiotic markers SCP3 and γ-H2AX. Additionally, the percentages of splenic CD4^+^ T and natural killer T (NKT) cell populations were significantly altered. In vitro, activated Dcaf12-deficient T cells displayed inappropriately stabilized MOV10 and increased levels of activated caspases. In summary, we identified MOV10 as a novel substrate of CRL4-DCAF12 and demonstrated the biological relevance of the DCAF12-MOV10 pathway in spermatogenesis and T cell activation.

## 1. Introduction

Selective protein degradation by the ubiquitin–proteasome system is essential for cellular homeostasis and regulation of diverse biological processes [1]. Substrate proteins are targeted for proteasomal degradation by covalent attachment of multiple ubiquitin molecules. The modification of a protein with ubiquitin is called ubiquitination and is catalyzed by a sequential action of three enzymes [2]. After activation by a ubiquitin-activating enzyme (E1), ubiquitin is transferred to the active site cysteine residue of a ubiquitin-conjugating enzyme (E2). In the last step, a ubiquitin ligase (E3) mediates ubiquitin transfer from E2 to a substrate protein [2]. Ubiquitin ligases recognize substrates via specific degradation signals (degrons), thus conferring selectivity to ubiquitination and subsequent protein degradation by the proteasome [3,4].

The human genome encodes more than 600 ubiquitin ligases, most of which belong to the RING domain family [5]. Among them, multisubunit cullin-RING ubiquitin ligases (CRLs) constitute the largest group. There are several cullins in mammals that act as scaffolds for the assembly of distinct CRL subfamilies. Cullin-4A (CUL4A) and cullin-4B (CUL4B), which exhibit an extensive sequence homology, assemble the cullin-RING ubiquitin ligase 4 (CRL4) subfamily [6]. The C-terminus of CUL4A/B interacts with RING finger protein RBX1 to recruit E2, while the N-terminus binds specific substrate receptors via adaptor DNA damage-binding protein 1 (DDB1). Substrate receptors, known as DDB1- and CUL4-associated factors (DCAFs), mediate substrate recognition and recruitment [7,8,9,10]. They typically consist of a WD-repeat domain responsible for substrate binding and docking to DDB1, which can be assisted by an α-helical H-box motif [11]. The modular assembly of CRLs provides the flexibility for targeting different substrates and is regulated by dynamic cycles of activation, inactivation, and substrate receptor exchanges [12]. CRLs are activated by covalent attachment of ubiquitin-like protein NEDD8 (neddylation) to cullins and inactivated by its rapid removal (deneddylation) [13,14,15]. Despite massive progress in uncovering the diversity of CRL4s, the substrates and function of most of them remain unknown or insufficiently characterized.

DDB1- and CUL4-associated factor 12 (DCAF12) was initially identified as a regulator of tissue growth and apoptosis in *Drosophila melanogaster* [16]. Furthermore, it was implicated in the Hippo pathway regulation [17] and showed to be essential for normal synaptic function and plasticity [18]. In placental mammals, DCAF12 has two close paralogs—DCAF12L1 and DCAF12L2 (protein sequence similarity ~70%) [19]. Although DCAF12L2 probably emerged by retrotransposition in the placental mammal ancestor, DCAF12L1 is present only in Euarchontoglires (a clade that includes rodents and primates) and seems to be a result of tandem duplication [20]. The expression pattern of DCAF12 paralogs differs from DCAF12, and it is unknown whether they assemble into functional CRL4. In human cells, DCAF12 regulates the stability of proteins ending in a twin-glutamic acid degron (C-terminal -EE degron) [21]. So far, only the regulation of melanoma antigen gene (MAGE) family members by DCAF12 has been studied [22]. Expression of MAGEs is normally restricted to male germ cells, but the genes are aberrantly reactivated in various cancers and drive tumorigenesis. In cancer cells, DCAF12 targets MAGE-A3 and MAGE-A6 for degradation in response to starvation [22]. However, the physiological function of DCAF12 in vertebrates remains unknown.

Moloney leukemia virus 10 (MOV10) is a highly conserved RNA helicase belonging to the UPF1-like group of helicase superfamily 1 [23,24]. MOV10 homologs have been found in plants (SDE3 in *Arabidopsis thaliana* [25]), nematodes (ERI-6/7 in *Caenorhabditis elegans* [26,27]), and insects (Armi in *Drosophila melanogaster* [28,29]). The vertebrate genome also encodes MOV10 paralog MOV10L1, which arose by gene duplication [30,31]. MOV10 and its homologs have an evolutionary conserved but enigmatic role in post-transcriptional gene silencing (RNA interference) and silencing of transposons, viruses, and recently duplicated genes [25,27,28,29,30,31,32,33,34]. These MOV10 activities are a crucial part of the host defense system across diverse species. MOV10 binds retrotransposon RNAs and is a potent inhibitor of retrotransposition [35,36,37,38,39]. Post-transcriptional reduction of retrotransposon transcripts [38] and inhibition of reverse transcription [37] were shown to be involved in the inhibition. However, the exact mechanism of retrotransposon restriction remains unclear [40,41]. Overexpression of MOV10 also inhibits replication and reduces infectivity of a wide range of exogenous retroviruses, including human immunodeficiency virus type-1 (HIV-1) [35,42,43,44,45]. Furthermore, MOV10 is an interferon-stimulated gene [46,47], which exhibits broad antiviral activity [46,48,49,50,51,52,53,54,55]. Apart from retrotransposon restricting and antiviral activities, MOV10 has an essential role in post-transcriptional gene regulation, especially in the microRNA (miRNA) pathway [56,57,58,59]. Human MOV10 predominantly binds to the 3′ UTR of mRNAs, in close proximity to miRNA recognition elements, and usually facilitates miRNA-mediated translational suppression [59]. Additionally, mouse MOV10 was shown to regulate miRNA biogenesis and was implicated in the splicing control [60]. Furthermore, MOV10 was suggested to facilitate nonsense-mediated mRNA decay [61] and implicated in Polycomb-mediated transcriptional silencing [62].

Here, we discovered that DCAF12 directly recognizes the C-terminal glutamic acid-leucine (-EL) degron of MOV10 and mediates its proteasome-dependent degradation. Additionally, we established *Dcaf12* knockout (KO) mice and found that DCAF12 controls the protein level of MOV10 during spermatogenesis and in T cells, especially after their activation. *Dcaf12* deficiency led to a decreased sperm count, dysregulation of immune cell populations, and increased splenocyte apoptosis after T cell activation. These observations highlight the biological importance of the DCAF12-mediated MOV10 degradation in vivo.

## 2. Results

### 2.1. Proteomic Analysis of DCAF12-Interacting Proteins

**Figure 1:**
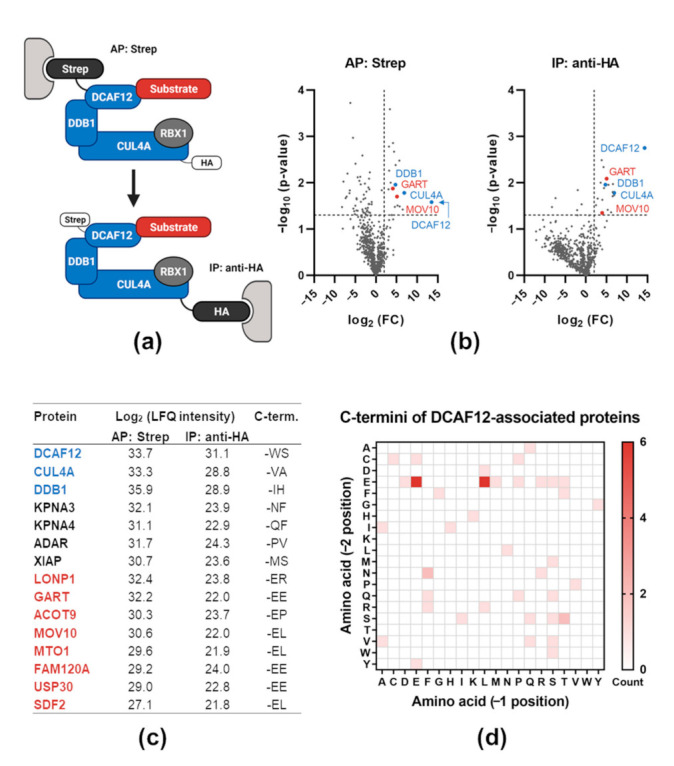
Proteomic analysis of DCAF12-interacting proteins. (**a**) Schematic representation of the tandem affinity purification strategy. StrepII-FLAG-tagged DCAF12 and HA-tagged CUL4A were co-expressed in HEK293T cells and subjected to affinity purification (AP) using Strep-TactinXT resin followed by immunoprecipitation (IP) using anti-HA magnetic beads. (**b**) CRL4^DCAF12^-associated proteins identified by liquid chromatography-tandem mass spectrometry (LC-MS/MS). The composition of purified complexes from both steps was analyzed by LC-MS/MS. Non-related ubiquitin ligase SCF^FBXL6^ was used as a control. Proteins that were significantly enriched with CRL4^DCAF12^ compared to SCF^FBXL6^ are in the upper right quadrant. (**c**) The top 15 hits obtained from LC-MS/MS analysis of CRL4^DCAF12^-associated complexes. Log_2_ (label-free quantitation (LFQ) intensities) from both purification steps and the last two C-terminal amino acids are shown. Components of CRL4^DCAF12^ are depicted in blue, putative substrates in red, other associated proteins in black. (**d**) Analysis of C-termini of CRL4-DCAF12-associated proteins. The top 50 hits were included in the analysis. The heat map shows the count of specific amino acid combinations at −2 (penultimate) and −1 (terminal) positions.

To reduce the presence of non-specific interactors of DCAF12, we adopted a tandem purification method to analyze the composition of cullin-based ubiquitin ligases. The procedure is based on sequential purification of a substrate receptor and a cullin scaffold protein. We validated this method using canonical ubiquitin ligase SKP1-CUL1-F-box protein (SCF)^β-TRCP^ and its interaction with various well-known substrates. These experiments showed significant enrichment in substrate isolation and, at the same time, reduction of non-specific binding (data not shown). Subsequently, we employed the same scheme to analyze potential substrates of multisubunit ubiquitin ligase CRL4^DCAF12^. We performed affinity purification (AP) of StrepII-FLAG-tagged DCAF12 (SF-DCAF12) followed by immunoprecipitation (IP) of hemagglutinin (HA)-tagged-CUL4A (HA-CUL4A) (Figure 1a) and analyzed the co-purified proteins by mass spectrometry. We obtained a similar number of “hits” from both purification steps, indicating that most DCAF12-bound proteins were associated with fully assembled CRL4^DCAF12^ (Figure 1b,c). We proceeded with an in silico analysis of the C-terminal amino acid sequence present in the DCAF12-bound proteins. In agreement with previous reports, proteins with two C-terminal glutamic acid residues (-EE) were enriched in DCAF12-associated proteins [21,22]. Moreover, we noticed an over-representation of glutamic acid-leucine residues at the extreme C-terminus (-EL), suggesting that the -EL motif might serve as an alternative DCAF12 degron (Figure 1d). Collectively, this approach allowed us to compile a list of putative DCAF12 substrates. Analysis of the physiological function of DCAF12-associated proteins showed a substantial presence of molecules involved in the immune, especially antiviral, response—e.g., MOV10, glycinamide ribonucleotide transformylase (GART), adenosine deaminase acting on RNA (ADAR), X-linked inhibitor of apoptosis protein (XIAP) [48,63,64,65]. To test whether these proteins could represent novel substrates of CRL4^DCAF12^, we selected MOV10 (the putative C-terminal-EL degron) and GART (the C-terminal-EE degron) for further validation.

### 2.2. DCAF12 Specifically Interacts with MOV10

**Figure 2:**
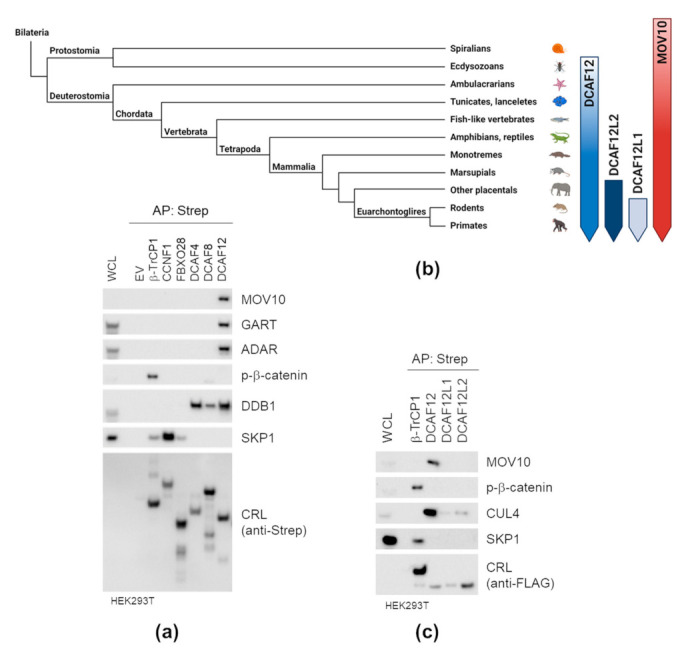
DCAF12 specifically interacts with MOV10. (**a**,**c**) Affinity purification (AP) of a panel of cullin-RING ubiquitin ligases (CRLs) (**a**) and DCAF12 and its paralogs (**c**). HEK293T cells were transfected with an empty vector (EV) or the indicated StrepII-FLAG-tagged CRL constructs and treated with MLN4924 for 6 h before harvesting. Forty-eight hours after transfection, cells were harvested, lysed, and whole-cell lysates (WCL) were subjected to AP with Strep-TactinXT resin. Eluates were immunoblotted with indicated antibodies. (**b**) A simplified evolutionary scheme illustrating the emergence of DCAF12 and its paralogs in bilaterians. DCAF12 is present in vertebrates and insects, DCAF12L2 in all placental mammals, and DCAF12L1 only in Euarchontoglires. Additionally, an ancient eukaryotic RNA helicase MOV10 is depicted.

To investigate whether the interaction between DCAF12 and MOV10 is specific, we expressed a panel of SF-F-box proteins and SF-DCAFs in HEK293T cells and performed small-scale AP of their protein complexes. We used the interaction between SCF^β-TRCP^ and its well-known substrate phospho-β-catenin, interactions between F-box proteins and their adaptor SKP1, and interactions between DCAF proteins and adaptor DDB1 as controls. We found that DCAF12 is the only substrate receptor able to interact with endogenous MOV10, GART and ADAR proteins (Figure 2a). Next, we performed subsequent interaction studies with mammalian paralogs of DCAF12 (Figure 2b). The interaction of DCAF12L1 and DCAF12L2 with CUL4 was weak, and the paralogs did not interact with MOV10 (Figure 2c). This indicated that they are not functionally redundant with DCAF12. Altogether, our AP experiments confirmed the specificity of the DCAF12-MOV10 interaction.

### 2.3. Nuclear Localization of DCAF12 Is Dispensable for Its Interaction with MOV10

**Figure 3:**
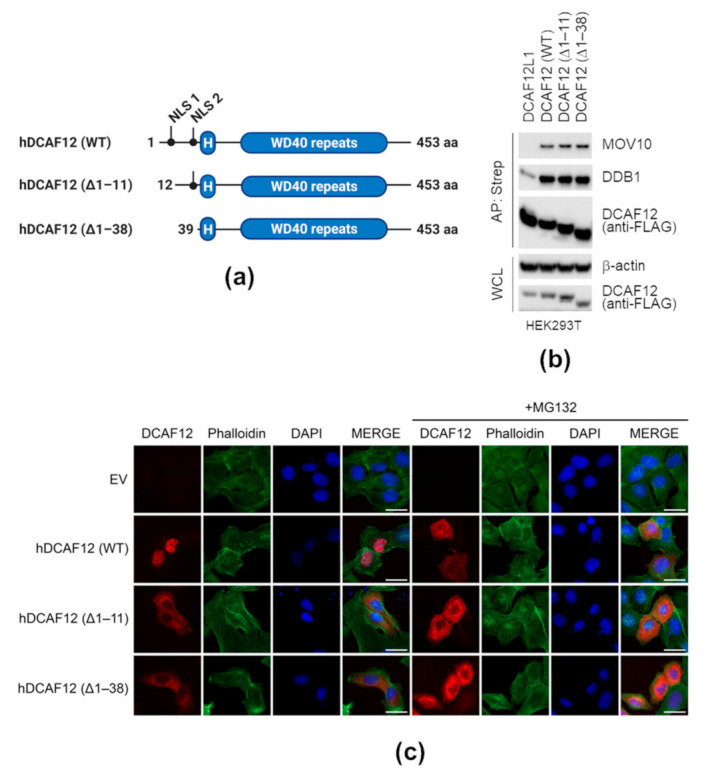
Nuclear localization of DCAF12 is dispensable for its interaction with MOV10. (**a**) Schematic representation of DCAF12 and its N-terminally truncated mutants. Two predicted nuclear localization signals (NLS) and H-box are highlighted. (**b**) Affinity purification (AP) of N-terminally truncated DCAF12 mutants. HEK293T cells were transfected with the indicated StrepII-FLAG-tagged DCAF12 constructs or DCAF12L1 as a control and treated with MLN4924 for 6 h before harvesting. Forty-eight hours after transfection, cells were harvested, lysed, and whole-cell lysates (WCL) were subjected to AP with Strep-TactinXT resin. Eluates were immunoblotted with indicated antibodies. (**c**) Subcellular localization of N-terminally truncated DCAF12 mutants. U2OS cells were transfected with an empty vector (EV) or the indicated StrepII-FLAG-tagged DCAF12 constructs and fixed 36 h later. DCAF12 was detected with the anti-Strep antibody. Phalloidin-iFluor 488 and diamidino-2-phenylindole (DAPI) were used to counterstain actin filaments and nuclei, respectively. Where indicated, cells were pre-treated for 6 h with MG132 before fixation. Scale bar, 30 μm.

MOV10 was shown to act in both the nucleus and cytoplasm [39,60,62]. To address whether DCAF12 nuclear localization is essential for interaction with MOV10, we disrupted two putative nuclear localization signals (NLSs) of DCAF12. Both are located near the N-terminus of DCAF12 preceding an α-helical H-box motif and a WD-repeat domain responsible for DDB1 and substrate binding [11]. Therefore, we prepared two N-terminally truncated mutants lacking the very N-terminal or both NLSs, and the variants were termed ∆1–11 or ∆1–38, respectively (Figure 3a). Importantly, the N-terminal truncation of DCAF12 did not affect its interaction with endogenous DDB1 and MOV10 (Figure 3b). However, we observed a decrease in MOV10 co-precipitated with SF-DCAF12^ΔNLSs^ in several cancer cell lines. In all cases, this reflected the increase in degradation of endogenous MOV10 by ectopically expressed DCAF12 proteins (Figure S1a,b,c). To investigate the subcellular localization of the DCAF12 truncated mutants, we transiently transfected U2OS cells with SF-DCAF12 and used an anti-Strep antibody for DCAF12 detection. Wild-type (WT) SF-DCAF12 localized to the nucleus, whereas SF-DCAF12^∆1–11^ and SF-DCAF12^∆1–38^ were both cytoplasmic, confirming the functionality of the first NLS (Figure 3c—left panel). These results were seemingly counterintuitive, as cytoplasmic proteins were predominantly present in DCAF12-co-purified complexes (Figure 1c). We thus hypothesized that the cytoplasmic location of DCAF12 could be transient and sensitive to degradation of its unknown cytoplasmic adaptor. Therefore, we treated SF-DCAF12-transfected cells with proteasome inhibitor MG132. This treatment led to significant accumulation of SF-DCAF12^WT^ in the cytoplasm (Figure 3c—right panel). Overall, these results showed that the NLSs of DCAF12 are indispensable for its interaction with MOV10. Additionally, localization of DCAF12 to the cytoplasm seems to be affected by an unstable and unknown factor regulating the subcellular localization of DCAF12.

### 2.4. MOV10 C-Terminal-EL Motif Is Necessary for Interaction with DCAF12

**Figure 4:**
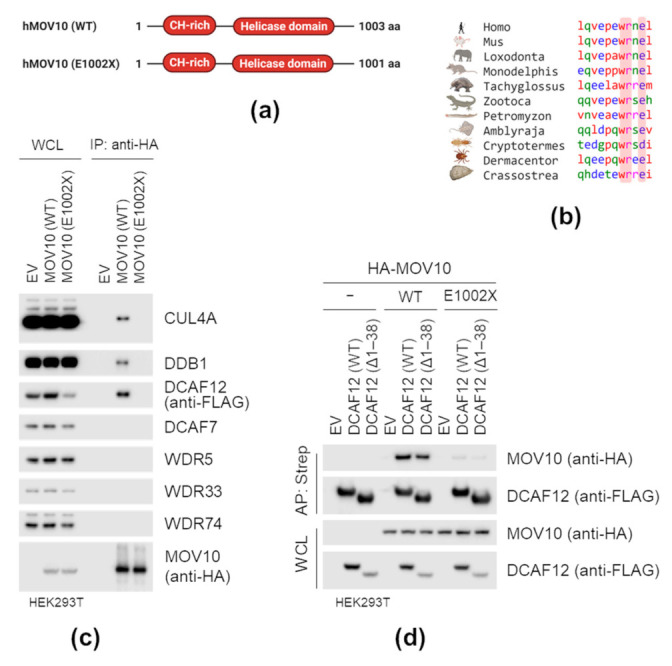
MOV10 C-terminal acidic motif is necessary for interaction with DCAF12. (**a**) Schematic representation of MOV10 and its E1002X mutant lacking the C-terminal -EL motif. (**b**) Multiple sequence alignment of the C-termini of MOV10 from indicated species. Sequences were aligned using Kalign [66]. (**c**) Immunoprecipitation (IP) of HA-tagged MOV10 (WT) and MOV10 (E1002X). HEK293T cells were co-transfected with StrepII-FLAG-tagged DCAF12 and either an empty vector (EV) or HA-tagged MOV10 (WT) and MOV10 (E1002X) constructs. MLN4924 was added for the last 6 h before harvesting. After lysis, whole-cell lysates (WCL) were subjected to anti-HA IP and immunoblotted as indicated. (**d**) Affinity purification (AP) of DCAF12 (WT) and DCAF12 (∆1–38) from HEK293T cells stably expressing HA-tagged MOV10 (WT) and MOV10 (E1002X). Parental and stably transduced HEK293 cells with doxycycline-inducible HA-tagged MOV10 (WT) and MOV10 (E1002X) constructs were transfected with an empty vector (EV) or StrepII-FLAG-tagged DCAF12 (WT) and DCAF12 (∆1–38). Twenty-four hours after transfection, cells were treated with doxycycline (DOX; 100 ng/mL) for another 24 h. MLN4924 was added for the last 6 h before harvesting. After lysis, whole-cell lysates (WCL) were subjected to AP and immunoblotted as indicated.

Human MOV10 ends with the C-terminal -EL motif. Interestingly, the motif is evolutionarily conserved and can be found in animals as divergent as oysters, termites, and lampreys (of note, it has diversified in insects and birds) (Figure 4b). To address whether the MOV10 C-terminal -EL motif represents a DCAF12 degron, we initially examined its necessity for interaction with DCAF12. We prepared a mutant form of MOV10 (E1002X) lacking the C-terminal -EL amino acid residues (Figure 4a) and tested its ability to interact with DCAF12. First, we co-expressed HA-tagged MOV10 WT or E1002X mutant with SF-DCAF12 in HEK293T cells and found that HA-MOV10, but not the E1002X mutant, co-immunoprecipitated SF-DCAF12, and endogenous CUL4A and DDB1. Additionally, HA-MOV10 could not co-immunoprecipitate any of the four additional WD-repeat domain proteins tested (Figure 4c). Then, we prepared HEK293T cell lines expressing HA-MOV10 or its E1002X mutant under the control of doxycycline-inducible promoter. Deletion of the last two amino acid residues did not affect the subcellular localization of MOV10 (Figure S2a), but prevented its interaction with SF-DCAF12^WT^ and SF-DCAF12^∆1–38^ (Figure 4d). Similarly, deletion of the C-terminal-EE motif prevented GART interaction with DCAF12 (Figure S2b). These findings provided further evidence that besides the canonical-EE degron, DCAF12 also directly recognizes the C-terminal-EL motif.

### 2.5. DCAF12 Controls the MOV10 Protein Level via Its C-Terminal Degron

**Figure 5:**
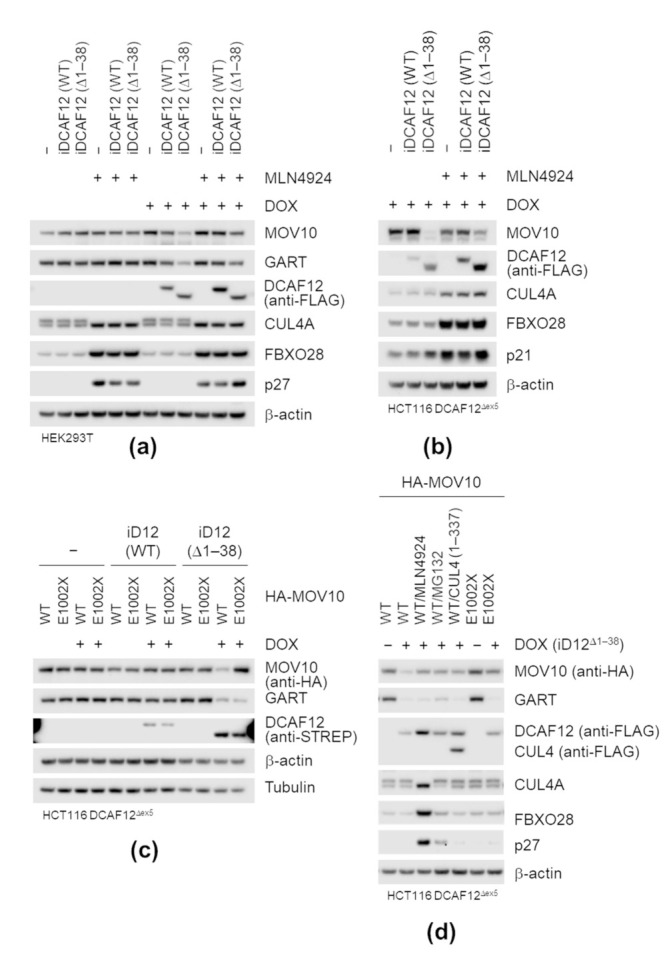
DCAF12 controls the MOV10 protein level via its C-terminal degron. (**a**,**b**) Induction of N-terminally truncated DCAF12 mutants in HEK293T (**a**) and HCT116^∆ex5^ (**b**) stable cell lines expressing StrepII-FLAG-tagged DCAF12 (WT) or DCAF12 (∆1–38) under the control of doxycycline-inducible promoter. Where indicated, cells were treated with doxycycline (DOX) for 48 h and MLN4924 overnight. Whole-cell lysates were subjected to immunoblotting with indicated antibodies. (**c**,**d**) Protein levels of transiently expressed MOV10 (WT) and MOV10 (E1002X) in HCT116^∆ex5^ stable cell lines expressing StrepII-FLAG-tagged DCAF12 (WT) or DCAF12 (∆1–38) under the control of doxycycline-inducible promoter (iD12). Cells were treated with doxycycline (DOX) for 48 h and transiently transfected with either HA-tagged MOV10 (WT) or (E1002X) 16 h before harvesting. Where indicated (**d**), cells were incubated with MLN4924 (O/N), MG132 (8 h), or co-transfected with dominant-negative FLAG-tagged CUL4A construct. Whole-cell lysates were subjected to immunoblotting with indicated antibodies.

Next, we investigated whether DCAF12 mediates MOV10 degradation in cancer cells. We previously noticed that overexpression of WT or cytoplasmic mutants of DCAF12 caused a decrease in the MOV10 protein level (Figure S1e). To test whether the decrease is CRL-dependent, we treated HEK293T cells harboring doxycycline-inducible DCAF12 expression constructs with neddylation inhibitor MLN4924. The induction of SF-DCAF12^∆1–38^ and, to some extent, also SF-DCAF12^WT^ decreased the MOV10 protein level, which was rescued by MLN4924. A similar pattern was observed for GART, another potential substrate of DCAF12; cullin-dependent substrates FBXO28, p27, p21 were used as controls for MLN4924 treatment (Figure 5a). We further generated *DCAF12* KO HCT116 cell lines (Figure S3a). Upon reintroduction of SF-DCAF12^∆1–38^, the MOV10 level significantly decreased. In agreement with the previous data from HEK293T cell lines, MLN4924 treatment rescued the effect of SF-DCAF12^∆1–38^ (Figure 5b). Interestingly, DCAF12 also underwent CRL-dependent degradation, as evident from the stabilization of DCAF12 by MLN4924 (Figure 5a,b,d and Figure S1d). No changes in *MOV10* mRNA levels were observed in either HEK293T or HCT116 cells (Figure S3b).

Finally, we expressed HA-MOV10^WT^ and HA-MOV10^E1002X^ in *DCAF12* KO HCT116 cell lines with inducible expression of SF-DCAF12 to test whether the deletion of C-terminal-EL amino acid residues was sufficient to prevent MOV10 degradation. Consistently with our previous results, the induction of SF-DCAF12^∆1–38^ led to elimination of endogenous GART and ectopically expressed HA-MOV10^WT^, but not HA-MOV10^E1002X^ (Figure 5c). Degradation of HA-MOV10^WT^ was abolished by MLN4924, MG132 (proteasome inhibitor), and co-expression of a dominant-negative form (amino acids 1–337) of CUL4 (Figure 5d), providing further evidence that the CRL4-dependent pathway mediated HA-MOV10 degradation. Taken together, our biochemical studies demonstrated that DCAF12 directly recognizes the C-terminal residues of MOV10 and mediates its proteasome-dependent degradation.

### 2.6. DCAF12 Controls the MOV10 Protein Level in Mice

**Figure 6:**
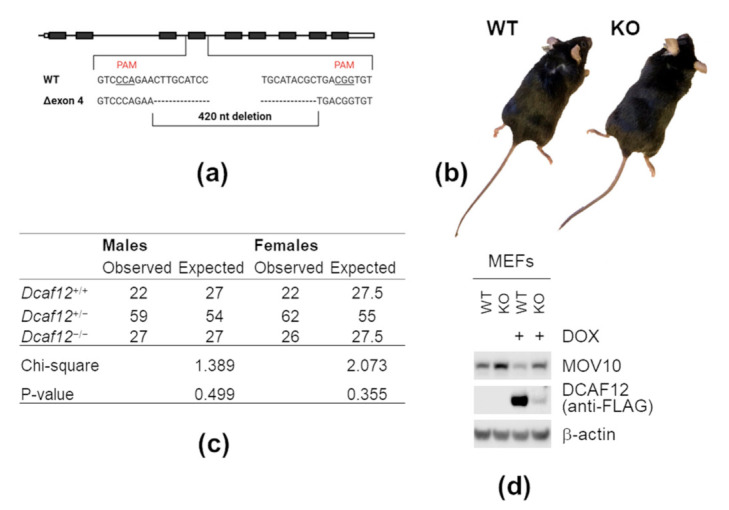
DCAF12 controls the MOV10 protein level in mice. (**a**) A schematic diagram of targeted disruption of the *Dcaf12* gene using a CRISPR/Cas9 genome-editing system in mice. Two single guide RNAs (sgRNAs) were designed to target introns flanking the exon 4 of the *Dcaf12* gene. The resulting deletion of exon 4 was 420-nt long. Protospacer adjacent motif (PAM) motives are underlined; dashes indicate deleted nucleotides. (**b**) Representative images of male mice of the indicated genotype. Mice did not display any apparent physical abnormalities. (**c**) Mice of all genotypes were born at expected Mendelian and gender ratios. The ratios were analyzed by Chi-square test, *p*-value > 0.05. (**d**) Mouse embryonic fibroblast (MEF) whole-cell lysates of the indicated genotype (*Dcaf12* WT or KO). MEFs were immortalized by CRISPR/Cas9-mediated inactivation of p19ARF. Subsequently, stable cell lines expressing StrepII-FLAG-tagged under the doxycycline-inducible promoter were established and treated with doxycycline (DOX) for 48 h. Whole-cell lysates were immunoblotted with indicated antibodies.

To investigate whether DCAF12 controls the MOV10 protein level in vivo, we generated mice with deletion of *Dcaf12* exon 4 (Figure 6a). Based on available expression data, this exon is constitutively used, and its deletion results in a frameshift leading to *Dcaf12* inactivation. This was supported by a significant decrease in transcript abundance, most probably due to the nonsense-mediated decay pathway (data not shown). *Dcaf12* KO mice were born at expected Mendelian ratios and manifested no apparent physical abnormalities (Figure 6b,c). Next, we established *Dcaf12* WT and KO mouse embryonic fibroblasts (MEFs). The protein level of MOV10 was increased in *Dcaf12* KO MEFs, which was reverted by reintroduction of SF-DCAF12 (Figure 6d and Figure S4a,b). This clearly showed that DCAF12 controls the protein level of MOV10 in mouse cells.

### 2.7. DCAF12 Controls the MOV10 Protein Level during Spermatogenesis

**Figure 7:**
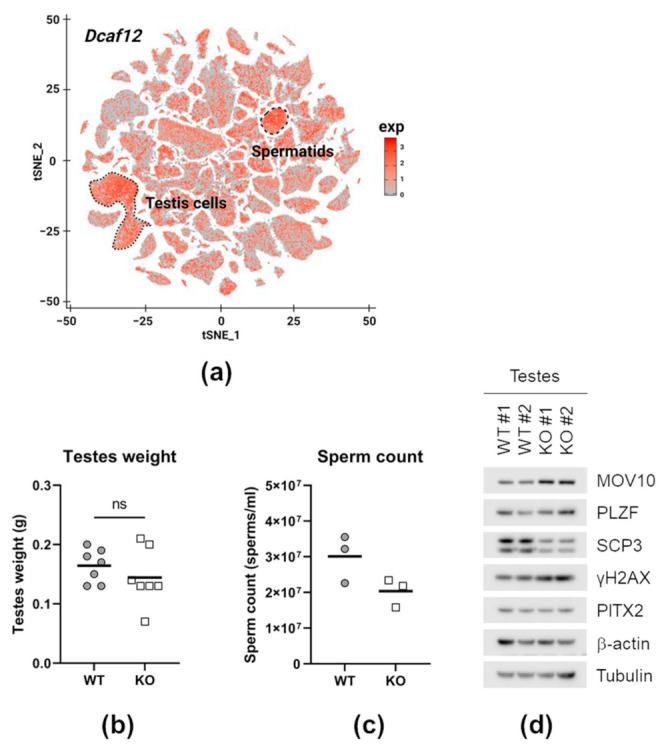
DCAF12 controls the MOV10 protein level during spermatogenesis. (**a**) *Dcaf12* expression projected on a t-distributed stochastic neighbor embedding (t-SNE) plot of single cells from publicly available mouse cell atlas (available at bis.zju.edu.cn/MCA; GEO accession number GSE108097) [67]. Testis cell and spermatid clusters are encircled. (**b**) Testes weight of 16-week-old *Dcaf12* WT and KO animals. Individual data points and means are shown (*n* = 7 per group). Statistical significance was assessed by an unpaired two-tailed t-test. *p*-value < 0.05 was considered significant; ns = not significant. (**c**) Sperm count of 16-week-old *Dcaf12* WT and KO animals. Individual data points and means are shown (*n* = 3 per group). (**d**) Testis whole tissue lysates from 16-week-old *Dcaf12* WT and KO mice (littermates, four animals). Testes without the tunica albuginea were lysed and subjected to immunoblotting with indicated antibodies.

The expression profile of *Dcaf12* indicated its prominent presence in the male reproductive system (Figure 7a). *Dcaf12* is highly expressed during late spermatogenesis (from pachytene spermatocytes). On the other hand, *Mov10* is mainly expressed in spermatogonia and certain subpopulations of pachytene spermatocytes (Figure S5). Although *Dcaf12* KO males manifested a moderate decline in sperm count, they were fertile. Additionally, we found no difference in the testes weight and morphology (Figure 7b,c and Figure S6a,b). Nevertheless, analysis of the whole-cell lysates from *Dcaf12* KO testes showed a reproducible increase in the protein level of MOV10 (Figure 7c and Figure S4c). Furthermore, meiotic marker SCP3 (synaptonemal complex protein 3) was slightly decreased and ɣ-H2AX (histone H2AX phosphorylated on serine 139) increased, suggesting disturbances during meiosis. At the same time, no changes in spermatogonial marker PLZF (promyelocytic leukemia zinc finger) and spermatocyte marker PITX2 (pituitary homeobox 2) were detected. Altogether, DCAF12 controls the MOV10 protein in the testes, and *Dcaf12* deficiency leads to a mild defect in spermatogenesis.

### 2.8. Dcaf12 Deficiency Leads to Dysregulation of Immune Cell Populations

**Figure 8:**
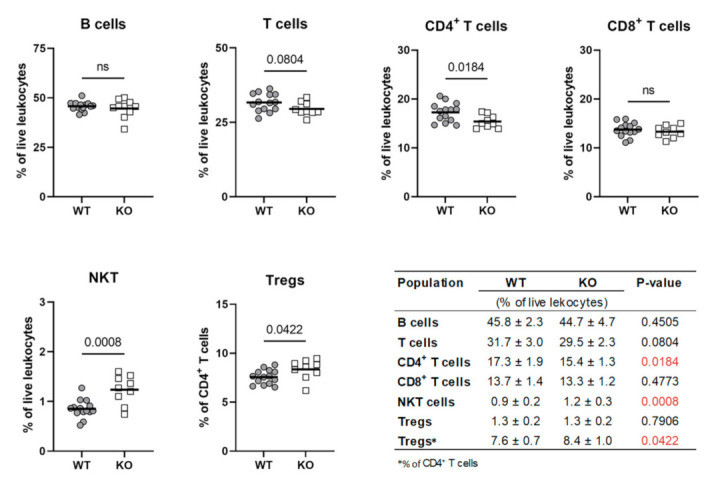
*Dcaf12* deficiency leads to dysregulation of immune cell populations. Quantification of immune cell populations in the spleen of 16–20-week-old *Dcaf12* WT and KO female mice. The populations were analyzed by flow cytometry, individual data points and group means are shown (*n* = 10–14 per group). Statistical significance was assessed by an unpaired two-tailed *t*-test. *p*-value < 0.05 was considered significant; ns = not significant. Means ± standard deviations and *p*-values are summarized in the table. Statistically significant *p*-values are highlighted in red.

A thorough analysis of the *Dcaf12* KO phenotype revealed alterations in immune cell populations in the spleen. Despite ubiquitous expression of *Dcaf12* and no differences in the spleen weight (Figure S7a,b), detailed immunophenotyping of splenocytes revealed that T cells were particularly affected in *Dcaf12* KO mice. Indeed, the percentage of CD4^+^ T cells was slightly but significantly reduced. Interestingly, the proportion of regulatory T cells (Tregs) was elevated within the CD4^+^ T cell population. Additionally, *Dcaf12* KO mice displayed a higher percentage of splenic NKT cells (Figure 8 and Figure S8). Collectively, these alterations in splenic populations indicated that DCAF12 might play an important role in the regulation of T cell development or homeostasis.

### 2.9. DCAF12 Controls the MOV10 Protein Level during T Cell Activation

**Figure 9:**
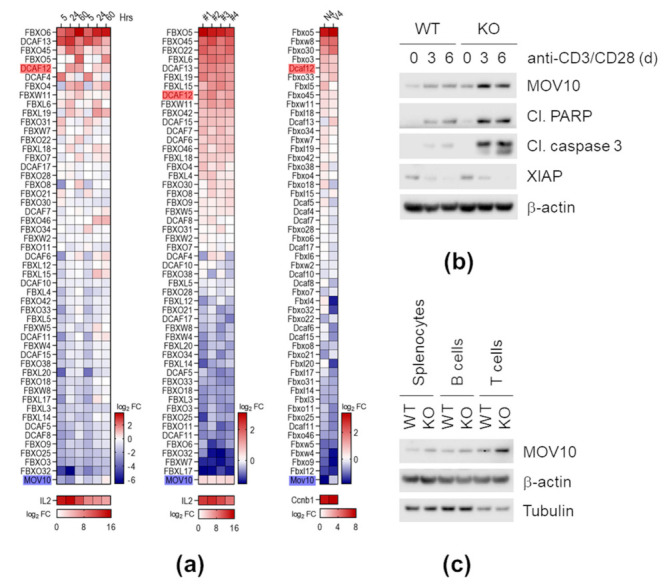
DCAF12 controls the MOV10 protein level during T cell activation. (**a**) Expression of SCF and CRL4 substrate receptors after T cell activation. Publicly available RNA-seq datasets were analyzed, and data were visualized as a heat map showing mean log_2_ fold changes (FC) of gene expression relative to the non-activated state. Left panel: Human naïve CD4^+^ T cells were activated with anti-CD3/CD28 or only anti-CD3 beads for the indicated time (GEO accession number GSE116697) [68]. Middle panel: Human naïve CD4^+^ T cells obtained from four donors were activated with anti-CD3/anti-CD28 beads for two days (GEO accession number GSE81810) [69]. Right panel: Mouse antigen-specific CD8^+^ T cells were activated for 48 h in the presence of high-affinity peptide N4 or low-affinity peptide V4 (right panel; GEO accession number GSE49929) [70]. Only substrate receptors appearing in all datasets are presented. Additionally, MOV10 and IL2 as a control are shown. DCAF12 is highlighted in red, MOV10 in blue. (**b**) Soluble fractions of lysates obtained from CD3/CD28-activated splenocytes. *Dcaf12* WT and KO splenocytes were stimulated with anti-CD3/CD28 Dynabeads for indicated times, lysed, and the soluble fractions were immunoblotted with indicated antibodies. (**c**) Soluble fractions of *Dcaf12* WT and KO splenocyte lysates. B and T cell-enriched populations were separated from freshly isolated splenocytes by negative selection using anti-CD45R (B220) antibody, lysed, and the soluble fractions were immunoblotted with indicated antibodies; Cl. PARP, cleaved form of poly (ADP-ribose) polymerase (PARP); Cl. caspase 3, cleaved form of caspase 3.

Following our observations on altered T cell populations, we further explored whether *Dcaf12* deficiency causes functional changes in T cells. A thorough analysis of publicly available RNA sequencing (RNA-seq) data showed that *Dcaf12*/*DCAF12* is significantly up-regulated upon T cell activation either by CD3/CD28 receptor crosslinking or T cell receptor (TCR)-specific peptide. Analysis of a panel of SCF and CRL4 substrate receptors, significantly expressed in both human and mouse T cells, revealed that *Dcaf12*/*DCAF12* is transcriptionally activated upon induction of TCR signaling in both organisms. *Mov10*/*MOV10* expression, on the other hand, was either not changed or down-regulated (Figure 9a). To examine whether DCAF12 mediates degradation of MOV10 during T cell activation, we stimulated mouse splenocytes with anti-CD3 and anti-CD28 (anti-CD3/CD28) antibody-coupled beads. Following stimulation, the MOV10 protein level was significantly increased in *Dcaf12* KO splenocytes, which exhibited increased apoptosis as determined by cleaved poly(ADP-ribose) polymerase (PARP) and cleaved caspase 3. On the other hand, caspase inhibitor XIAP, another DCAF12-associated protein, was strongly down-regulated upon T cell activation in both *Dcaf12* WT and KO splenocytes (Figure 9b). In our setting, the *MOV10* mRNA levels increased upon stimulation to the same extent in *Dcaf12* KO and WT splenocytes (Figure S9a). The activation of *Dcaf12* KO splenocytes seemed to be unchanged (Figure S9b). Next, we examined the MOV10 protein level in splenocytes treated with different stimuli. Stimulation with poly(I:C) or lipopolysaccharides (LPS) caused comparable up-regulation of MOV10 between *Dcaf12* KO and WT splenocytes, suggesting that the DCAF12-dependent down-regulation of the MOV10 protein level occurs specifically after T cell activation (Figure S10a,b). As we noticed a slight but reproducible increase in the MOV10 protein level in *Dcaf12* KO splenocytes, even without stimulation with anti-CD3/CD28 beads, we investigated in which splenic cell populations the protein was up-regulated. Therefore, we prepared lysates from T and B cell-enriched splenic populations and observed a pronounced increase of MOV10 in the T cell-enriched sample (Figure 9c). Taken together, DCAF12 controls the MOV10 protein level and inhibits caspase 3 during T cell activation.

## 3. Discussion

The diversity of C-terminal degron pathways and their biological functions are only currently being elucidated. We focused on DCAF12, which has recently been established as one of the E3 ubiquitin ligases targeting C-terminal degrons, specifically the -EE motif [21]. Here, we showed that DCAF12 also mediates degradation of proteins bearing the C-terminal -EL motif. Furthermore, it might target other C-terminal -EX motives (X stands for any amino acid), as proteins with glutamic acid at the penultimate (−2) position and different C-terminal residues were also identified in our MS data. Functional classification of DCAF12-associated proteins revealed a substantial presence of those involved in the immune and antiviral responses (e.g., MOV10, GART, ADAR, XIAP [48,63,64,65]), raising an intriguing possibility that the DCAF12 pathway might regulate activity or homeostasis of the immune system. Among DCAF12 potential substrates, proteins localizing to the cytoplasm, mitochondria, or secretory pathway were significantly enriched. This suggests that beyond the regulation of cytoplasmic proteins, DCAF12 might also contribute, together with other C-terminal degron pathways [71], to elimination of secretory or mitochondrial proteins mislocalized to the cytoplasm.

As DCAF12 recognizes degrons constitutively present in proteins, the question of how it is regulated remains to be answered. The absence of reliable anti-DCAF12 antibodies thwarted our effort to monitor ubiquitously expressed endogenous DCAF12 and compelled us to use either inducible or overexpression systems. DCAF12 localized mainly to the nucleus, and this localization depended on DCAF12 N-terminal NLS. We hypothesize that DCAF12 is sequestrated to the nucleus and only transiently localized to the cytoplasm, where it gains access to the potential substrates. An unstable and not yet recognized cytoplasmic factor might restrict access of DCAF12 to the nucleus under certain conditions. This is supported by our observation of DCAF12 accumulation in the cytoplasm after proteasome inhibition. Alternatively, the DCAF12 localization could be regulated via post-translational modification. Serine 15, located in the vicinity of the first NLS, was repeatedly shown to be phosphorylated [72]. Several papers identified this event in immune cells or during viral infection [73,74]. Interestingly, adjacent serine 13 was identified to be phosphorylated upon stimulation of serum-starved cells with insulin [75]. Moreover, DCAF12 was shown to target MAGE-A3/6 for degradation in serum-starved cells, and this degradation was rescued by insulin [22]. Therefore, we speculate that a pathway downstream of the insulin receptor (e.g., the Akt signaling pathway) might regulate the subcellular localization of DCAF12 and, consequently, its function. Additionally, the DCAF12 function might be controlled by its stability. We repeatedly noticed an increase in the DCAF12 protein level after inhibition of CRL neddylation (MLN4924) and proteasome (MG132) and fast DCAF12 degradation after inhibition of protein synthesis (cycloheximide treatment). The instability of DCAF12 itself might be the reason we could not see changes in the MOV10 stability in the cycloheximide chase assay. Of note, constitutive cytoplasmic mutants of DCAF12 were more stable than WT DCAF12 (data not shown). The instability of DCAF12 might be caused by the unavailability of DCAF12 substrates (especially in the nucleus), as the absence of CRL substrates often leads to CRL self-ubiquitination and degradation [76,77]. Alternatively, other E3 ubiquitin ligases might control the DCAF12 pathway. A well-known example of this is the degradation of CDT2 (a DCAF protein) by SCF^FBXO11^ [78]. Altogether, we hypothesize that the DCAF12 pathway is controlled by subcellular localization and stability of DCAF12.

Our study comprehensively demonstrates that DCAF12 mediates degradation of MOV10 both in human and mouse cells. As discussed above, DCAF12 is mainly nuclear in human cancer cells, while MOV10 is dominantly cytoplasmic. Nevertheless, the nuclear localization of MOV10 was reported in human primary fibroblasts [62]. In mice, MOV10 was observed both in the cytosol and in the nucleus, depending on the cell type and developmental stage [39,60]. The different subcellular localization of DCAF12 and MOV10 in human cancer cells might explain why we could not reliably observe an increased MOV10 protein level and stability after DCAF12 depletion using small inhibitory (si) RNA or in *DCAF12* KO HCT116 cells (data not shown). On the other hand, MOV10 was efficiently degraded upon induction or overexpression of DCAF12^∆1–38^ and, to some extent, also by DCAF12^WT^. In mice, MOV10 was up-regulated in *Dcaf12* KO MEFs, and reintroduction of DCAF12 rescued this effect. Furthermore, *Dcaf12* deficiency led to up-regulation of MOV10 in several tissues, demonstrating that DCAF12-mediated degradation of MOV10 is biologically relevant.

Using our *Dcaf12* KO mice, we found that DCAF12 controls the MOV10 protein levels during spermatogenesis. In murine testes, *Mov10* is mainly expressed in spermatogonia, where it was shown to regulate miRNA biogenesis and was implicated in the splicing control [60]. However, its protein level sharply declines in pachytene spermatocytes [60], in which *Dcaf12* is highly expressed. These data suggest that DCAF12 could regulate MOV10 during late spermatogenesis, but this hypothesis needs to be further experimentally validated. Although our histological examination of tubular sections did not reveal any apparent alterations in spermatogenesis, we detected a slight decline in the sperm count. It was accompanied by a decrease in meiotic marker SCP3 and an increase in DNA damage marker ɣ-H2AX. This observation might indicate meiotic disturbances, which often lead to decreased sperm count [79,80]. However, a broader analysis is required to elucidate at which stage of spermatogenesis the defect happens and whether the up-regulation of only MOV10 or other DCAF12 substrates is responsible. As MOV10 participates in the miRNA pathway [60], essential for spermatogenesis [81,82,83], MOV10 up-regulation in the testes of Dcaf12-deficient mice could potentially impact the pathway. Additionally, elevated MOV10 could also affect the restriction of retrotransposons, which is an evolutionarily conserved function of MOV10 [25,27,28,29,30,31,32,33,34]. Although this function is attributed to MOV10L1 in the germline [30,31], the contribution of MOV10 to the restriction of retrotransposons in the testes remains to be clarified. Of note, a specific subset of endogenous retroviruses is required to drive the transcription of germline genes in late spermatogenesis [84]. As MOV10 was also implicated in transcriptional silencing [62], excessive retrotransposon silencing by increased MOV10 might be detrimental as well. Altogether, we propose that DCAF12-mediated degradation of MOV10 and possibly other substrates might affect spermatogenesis, but the underlying mechanism has yet to be elucidated.

Furthermore, we discovered that DCAF12 controls the MOV10 protein level in T cells, especially after their activation. MOV10 is involved in the miRNA pathway, which has a central role in regulating the development, homeostasis, and function of immune cells [85,86]. Detailed immunophenotyping of the splenic immune cell populations in *Dcaf12* KO animals indicated a significant defect in the T cell lineage. Although other DCAF12 substrates can be responsible for the phenotype, we observed elevated MOV10 in T cells. MicroRNA pathway-deficient T cells exhibit increased apoptosis, primarily upon stimulation, and reduction of the percentage of T cells in the peripheral compartments such as the spleen [87,88,89], both of which we have seen. The control of MOV10 by DCAF12 was especially pronounced during T cell activation, during which *Dcaf12* mRNA is up-regulated. T cell activation induces dramatic changes in the miRNA repertoire, which causes global post-transcriptional reprogramming and enables precise immune responses [90,91,92]. MOV10- and miRNA pathway-associated protein AGO2 is degraded following T cell activation [90]. We thus speculate that DCAF12-mediated degradation of MOV10 might contribute to the changes in the miRNA repertoire in T cells.

Beyond its role in the miRNA pathway, MOV10 restricts retrotransposons, including retroviruses [35,36,37,38,40,41,42,43,44,45]. Most retrotransposons are silenced, but some are transcriptionally active or contribute to the normal regulation of gene expression in immune cells [93,94]. Additionally, it was already demonstrated that exposure of immune cells to microbial products, e.g., LPS or poly(I:C), triggers expression of distinct retrotransposons and endogenous retroviruses [95]. Our analysis of publicly available data (not shown) unveiled that this is often accompanied by increased expression of *Mov10* and down-regulation of *Dcaf12* mRNA, for example, after stimulation with LPS or Toll-like receptor 2 (TLR2) agonists. In support of this, we observed the up-regulation of MOV10 in the splenocytes treated with various TLR agonists. Therefore, we speculate that MOV10 could be dynamically regulated after exposure to different pathogen-associated molecular patterns. Such up-regulation of MOV10 could protect cells from increased expression of retrotransposons, including endogenous retroviruses. Here, we showed that *Dcaf12* is up-regulated and controls the MOV10 protein level when splenocytes are activated with anti-CD3/CD28 antibody-coupled beads. Interestingly, CD3/CD28 receptor crosslinking, but not LPS stimulation, leads to reactivation of latent HIV-1 in resting CD4^+^ T cells [96,97]. Based on these observations, we suggest that the down-regulation of the MOV10 protein level by DCAF12 in activated T cells creates a “window of opportunity” for HIV-1 to evade the host retrovirus surveillance pathways. The unrestricted retrotransposon activation immediately after TCR stimulation could pre-activate antiviral innate immunity pathways in T cells, and thus represent a novel mechanism of cellular self-defense against viruses. This hypothesis needs to be validated, and *Dcaf12* KO mice represent an elegant model for such a study.

DCAF12 was previously implicated in several immunological processes in humans. In the peripheral blood, the decreased expression of *DCAF12* (also known as *WDR40A* in clinical articles) precedes the onset of post-transplantation organ rejection [98,99]. Additionally, *DCAF12* was associated with the development of intestinal Behçet’s disease, chronic inflammatory disorder [100]. Our data also revealed dysregulation of various T cell populations in the spleen of *Dcaf12* KO animals, which indicates a defect in T cell development or homeostasis. Moreover, *Dcaf12* deficiency affected the physiological status of activated T cells. As distinct T cell subpopulations are involved in diverse immune processes, *Dcaf12* KO animals offer a fertile ground for new discoveries, especially but not only in the field of transplantation immunology and research of inflammatory disorders.

## 4. Materials and Methods

### 4.1. DNA Constructs

Human MOV10, GART, CUL4A, DCAF12, DCAF12L1, and DCAF12L2 were PCR-amplified from a cancer cell line-derived cDNA library and inserted into modified pcDNA3.1 vectors containing the HA-tag (pcDNA3.1-HA) or the previously described [101] StrepII-FLAG-tag for N-terminal fusion (pcDNA3.1-SF). Truncated versions of DCAF12 (Δ1–11, Δ1–38), MOV10 (E1002X), and GART (E1009X) were prepared in a similar way using corresponding pcDNA3.1 constructs as templates. Dominant-negative CUL4A was prepared by inserting the N-terminal part (aa 1–337) of CUL4A into the pcDNA3.1-FLAG-HA backbone. Lentiviral pTRIPZ-DCAF12 plasmids were constructed by PCR amplification of cDNA from the corresponding pcDNA3-NSF-DCAF12 plasmids and cloning into the pTRIPZ backbone (Dharmacon, Lafayette, CO, USA) using AgeI-XhoI/SalI restriction sites. pSBtet-Pur-NSF-DCAF12 and pSBtet-Pur-HA-MOV10 plasmids were prepared similarly by PCR amplification of the NSF-DCAF12 or HA-MOV10 sequence and its insertion into pSBtet-Pur (Addgene plasmid # 60507) using SfiI sites. All plasmids were verified by sequencing. Primers are listed in Table S1.

### 4.2. Mice

*Dcaf12* KO mice (∆exon 4) were generated in a C57BL/6N background using the CRISPR/Cas9 genome-editing system. For this purpose, the Cas9 protein and gene-specific sgRNAs (Integrated DNA Technologies) were used for zygote electroporation using a protocol described previously [102]. Single guide RNA sequences with the PAM in bold (3′end) were as follows:

sgRNA target 1: ATAGGGCATGCATACGCTGA**CGG**

sgRNA target 2: GAGGCCGAGGATGCAAGTTC**TGG**

The genome editing was confirmed by PCR amplification in the founder mouse with the primers listed in Table S1. Generation of *Dcaf12* KO mice (MGI:1916220; *Dcaf12em1(IMPC)Ccpcz* allele) was part of the International Mouse Phenotyping Consortium (IMPC) project, and animals are available upon request. The mutant allele was backcrossed for two generations to C57BL/6N mice. Animals were maintained in a controlled, specific pathogen-free environment at the animal facility of the Czech Center for Phenogenomics in Vestec, Institute of Molecular Genetics of the Czech Academy of Sciences, Czech Republic.

### 4.3. Cell Lines and Primary Cells

All cell lines and primary cells were cultured in a humidified incubator at 37 °C and 5% CO_2_. HEK293T, HCT116, U2OS, HeLa (American Type Culture Collection (ATCC), Manassas, VA, USA), and MEFs were grown in Dulbecco’s Modified Eagle’s Medium (DMEM; Sigma-Aldrich, St. Louis, MO, USA) supplemented with 10% fetal bovine serum (FBS; Biosera, Nuaille, France), 100 IU/mL penicillin, 100 µg/mL streptomycin, and 40 µg/mL gentamicin. BJAB (ATCC, Manassas, VA, USA) and mouse primary splenocytes were grown in RPMI-1640 (Sigma-Aldrich, St. Louis, MO, USA) supplemented with FBS and antibiotics as described above.

The DCAF12 KO (∆exon 5) HCT116 cell line was generated using CRISPR/Cas9 genome editing as described previously [103] with slight modifications. Single guide RNA target sequences with the PAM motif in bold (3’end) were as follows:

sgRNA target 1: AAGCCCTCTTTGGCGTCTGT**GGG**

sgRNA target 2: AAGCAAGAGGAATACACGTC**AGG**

Briefly, targeting sequences were cloned into the pXPR_001 vector (Addgene plasmid #49535). Selected cell lines were transfected with a mixture of plasmids and shortly selected with puromycin (1 µg/mL). Single-cell clones were generated, and PCR confirmed the correct genome editing with the primers listed in Table S1.

Stable HCT116, HEK293T, HeLa, BJAB cell lines expressing StrepII-FLAG-tagged DCAF12 (WT), DCAF12 (∆1–11), or DCAF12 (∆1–38) under the control of doxycycline-inducible promoter were generated using the lentiviral system as described previously [104]. Briefly, lentiviral particles were produced in HEK293T cells by co-transfecting pCMV-VSV-G (Addgene plasmid #8454), pCMV-dR8.2 (Addgene plasmid #8455) with either pTRIPZ-DCAF12 (WT), pTRIPZ-DCAF12 (∆1–11), or pTRIPZ-DCAF12 (∆1–38). Forty-eight hours after transfection, viral supernatant was collected, filtered, and added to target cells at a 1:1 ratio in the presence of 10 μg/mL Polybrene (Sigma-Aldrich, St. Louis, MO, USA). The following day, the cell media was changed, and cells were subjected to puromycin (1 µg/mL) selection.

Stable HEK293 and HCT116 cell lines expressing HA-tagged MOV10 (WT) or MOV10 (E1002X) were prepared using the Sleeping Beauty transposable system, as described previously [105]. Briefly, cells were co-transfected with pSB100X and pSBtet-Pur-MOV10 (WT) or pSBtet-Pur-MOV10 (WT) vectors. Twenty-four hours after transfection, cells were subjected to puromycin selection (1 µg/mL).

Mouse embryonic fibroblasts were derived from embryonic day (E) 13.5 mouse embryos using a previously described protocol [106] and immortalized by two different approaches. Primary MEFs were transduced as described above with pLenti CMV/TO SV40 Small and Large T antigen (w612-1) (Addgene plasmid #22298). Alternatively, primary MEFs were immortalized by the loss of p19ARF [107]. To inactivate p19ARF, the CRISPR-Cas9 genome-editing technology and px330-Cas9-p19Arf sgRNA vectors (kindly provided by Prof Tomas Stopka) were used. Immortalized MEFs were co-transfected with pSB100X and pSBtet-Pur-DCAF12 as described above to generate stable cell lines expressing FLAG-Twin-Strep-tagged DCAF12 under the control of doxycycline-inducible promoter. The concentration of puromycin used for selection was 5 µg/mL.

Single-cell splenocyte suspensions were prepared by mechanical dissociation of spleen tissue and filtered through 70 μm cell strainers. Red blood cells were depleted by ammonium-chloride-potassium (ACK) lysis buffer (150 mM NH_4_Cl, 10 mM KHCO_3_, 0.1 mM Na_2_EDTA; pH 7.2–7.4).

### 4.4. Cell Treatments and Transient Transfection

Cells were treated with the following inhibitors: 1 µM MLN4924 (Santa Cruz Biotechnology, Dallas, TX, USA), 10 µM MG132 (MedChem Express, Monmouth Junction, NJ, USA), 100 µg/mL cycloheximide (Sigma-Aldrich, St. Louis, MO, USA), 1 µg/mL doxycycline hyclate (Sigma-Aldrich, St. Louis, MO, USA), 10 µg/mL Poly(I:C) (Invivogen, San Diego, CA, USA), and 5 µg/mL LPS (Sigma-Aldrich, St. Louis, MO, USA).

Plasmid transient transfections were carried out using the polyethylenimine (PEI; Linear, MW 25000, Transfection Grade) transfection reagent (Polysciences, Valley Road, Warrington, PA, USA) as described previously [108].

### 4.5. Cell Lysis, Immunoprecipitation, and Affinity Purification

Cells and testes were lysed in 1× lysis buffer (150 mM NaCl, 50 mM Tris pH 7.5, 1 mM EDTA, 0.4% Triton-X, 2 mM CaCl_2_, 2 mM MgCl_2_, 5 mM NaF) supplemented with 1 mM Na_3_VO_4_, protease inhibitors (10 µM TLCK, 10 µM TPCK, 0.8 mM PMSF), 1 mM DTT (except for lysates intended for IP), and 125 U/mL benzonase (Santa Cruz Biotechnology, Dallas, TX, USA). For testes lysis, tunica was first mechanically removed, and the rest of the tissue was mixed with lysis buffer and disrupted by TissueLyser (Qiagen, Hilden, Germany). After incubation on ice, native lysates were cleared by centrifugation and used for IP or AP. Alternatively, lysates were mixed 1:1 with 2% SDS in 50 mM Tris-HCl (pH 8), heated for 5 min at 95 °C, cleared by centrifugation, and further processed for immunoblotting. If only the soluble fraction was to be analyzed, benzonase was omitted in the lysis buffer, and the soluble fraction was finally obtained after centrifugation.

HA-tagged proteins were immunoprecipitated using anti-HA magnetic beads (Thermo Fisher Scientific, Waltham, MA, USA), while Twin-Strep-tag recombinant proteins were purified using Strep-TactinXT Superflow resin (IBA Lifesciences, Göttingen, Germany). Immunoprecipitated proteins were eluted by adding 1× Bolt LDS Sample Buffer (Thermo Fisher Scientific, Waltham, MA, USA) mixed with β-mercaptoethanol and subsequent incubation at 95 °C for 5 min. Purified proteins were eluted by 1× Buffer BXT (IBA Lifesciences, Göttingen, Germany). Eluates were subsequently mixed with Bolt LDS Sample Buffer (Thermo Fisher Scientific, Waltham, MA, USA) supplemented with β-mercaptoethanol (final concentration 2.5%) and incubated at 95 °C for 5 min.

### 4.6. Immunoblotting

Proteins were separated in NuPAGE 4–12% Bis-Tris gels (Thermo Fisher Scientific, Waltham, MA, USA) and transferred to Amersham Hybond P 0.45 μm PVDF membrane (GE Healthcare, Chicago, IL, USA). Membranes were blocked with 5% milk (Santa Cruz Biotechnology, Dallas, TX, USA), incubated with indicated primary antibodies diluted in 3% BSA (Applichem, Darmstadt, Germany) in TBS-T overnight at 4 °C and appropriate HRP-conjugated secondary antibodies (Cell Signaling Technology, Danvers, MA, USA) diluted in 5% milk in TBS-T, and developed with either WesternBright ECL HRP substrate (Advansta, San Jose, CA, USA) or SuperSignal West Femto Maximum Sensitivity Substrate (Thermo Fisher Scientific, Waltham, MA, USA). Antibodies are listed in Table S2.

### 4.7. Tandem Affinity Purification and Mass Spectrometry

HEK293T cells were co-transfected with StrepII-FLAG-tagged DCAF12 and HA-tagged CUL4A, grown for 48 h, harvested, and lysed as described above. In the first step, Strep-TactinXT Superflow resin (IBA Lifesciences, Göttingen, Germany) was used to purify proteins associated with StrepII-FLAG-tagged DCAF12. In the second step, eluates from the previous step were used for immunoprecipitation of HA-CUL4A-associated proteins. Eluates from both steps were analyzed by LC-MS/MS at the Proteomics facility of Biotechnology and Biomedicine Center of the Academy of Sciences and Charles University (BIOCEV) in Vestec, Czech Republic. Further details are provided in Supplementary methods.

### 4.8. Immunofluorescence Microscopy

Cells were grown on glass coverslips and fixed with 4% PFA for 20 min, permeabilized with 0.2% Triton-X-100 in PBS for 10 min and blocked in 3% BSA in PBS with 0.1% Triton-X-100 for 1 h. Coverslips were incubated with anti-StrepII Tag antibody (Novus Biologicals, Littleton, CO, USA; #NBP2-43735, 1:1000) for 2 h. Alexa Fluor555-conjugated goat anti-mouse IgGs (Abcam, Cambridge, UK; ab150110, 1:1000) was used as a secondary antibody. Phalloidin-iFluor 488 Reagent (Abcam, Cambridge, UK; ab176753, 1:1000) was used to stain F-actin. Nuclei were stained by DAPI. Slides were mounted with ProLong Gold Antifade Mountant (Thermo Fisher Scientific, Waltham, MA, USA). Image acquisition was performed using a Zeiss Axio Imager.Z2 microscope equipped with ZEN software (Zeiss, Oberkochen, Germany).

### 4.9. Epididymal Sperm Count

Both caudal epididymides of 16-week-old Dcaf12 WT and KO animals were collected into 1 mL of PBS, minced with forceps, and incubated for 10 min. Sperm counts were determined using the Bürker chamber.

### 4.10. Immunophenotyping of Splenocytes; T Cell Enrichment and Activation

Immune cell subpopulations in the spleen were analyzed by flow cytometry according to the standard IMPC immunophenotyping protocol [109].

T cells were enriched by negative selection using biotinylated anti-CD45R (B220) antibody (BioLegend, San Diego, CA, USA; #103203). Briefly, freshly isolated splenocytes were incubated for 1 h in a cell culture plate coated with the CD45R (B220) antibody. Medium with unattached cells (further referred to as a T cell-enriched population) was then removed and transferred to the second plate. The cells remaining in the first plate were considered a B cell-enriched population. B cell depletion was confirmed using flow cytometry analysis. According to the manufacturer’s instructions, T cells were stimulated for indicated times with Dynabeads Mouse T-Activator CD3/CD28 (Thermo Fisher Scientific, Waltham, MA, USA).

### 4.11. Statistical Analysis and Data Visualization

Differences between *Dcaf12* WT and KO groups were evaluated by unpaired two-tailed t-test, while chi-square test was used to assess the mouse offspring ratios. *p*-value < 0.05 was considered significant. Individual data and means are shown in the figures. The statistical analyses were performed using GraphPad Prism 9.0 (GraphPad Software, San Diego, CA, USA). Illustrations were created with BioRender.com.

## 5. Conclusions

**Figure 10:**
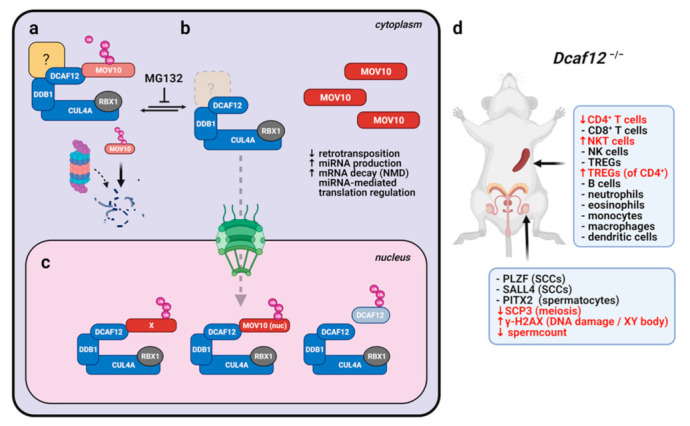
Model of DCAF12-dependent regulation of the MOV10 protein and its physiological consequences. (**a**) Unknown unstable factor sequesters the DCAF12 protein in the cytosol, where it regulates the MOV10 protein level. (**b**) Degradation of this factor enables DCAF12 relocation into the nucleus, where DCAF12 targets its nuclear substrates. (**c**) Depletion of cytosolic DCAF12 leads to MOV10 stabilization, which might enhance its roles in the regulation of miRNA and antiviral functions. NMD: non-sense-mediated decay. (**d**) *Dcaf12*-deficient mice are viable. However, inappropriate stabilization of DCAF12 substrates leads to slight deregulation of cellular subpopulations in the testes and spleen.

We identified MOV10 as a novel substrate of CRL4^DCAF12^ (Figure 10). Specifically, DCAF12 directly recognizes the C-terminal acidic degron of MOV10 and targets MOV10 for proteasome-dependent degradation. Notably, we established a *Dcaf12* KO mouse model and showed that DCAF12 controls the protein level of MOV10 during spermatogenesis and after T cell activation. MOV10 is crucial for retrotransposon restriction and miRNA-mediated post-transcriptional regulation of gene expression. Therefore, we suggest that DCAF12 is critical for fine-tuning the protein level of MOV10 to precisely balance the control of retrotransposons and viruses on the one hand and the miRNA-regulated transitions (e.g., from resting to activated T cells) on the other. *Dcaf12* KO mice might serve as a valuable model of the role of endogenous retroviruses and miRNA pathway in the control of gene expression and cellular differentiation during mammalian development. Furthermore, we have provided evidence for the role of DCAF12 in the immune system, highlighting the clinical importance of the DCAF12 pathway.

## Data Availability

All experimental data are provided within the publication or its Supplementary files. Additionally, the following public databases were used: bis.zju.edu.cn/MCA, tabula-muris.ds.czbiohub.org doi.org/10.17632/kxd5f8vpt4.1, www.ncbi.nlm.nih.gov/gds, www.ilincs.org/apps/grein (All accessed on 15 March 2021). The accession numbers are indicated in the text or figure legends. Cross-sections of Dcaf12 WT and KO testes are openly available in Mendeley data repository at doi:10.17632/h4g7ctf2wc.1.

## Supplementary Materials

The following are available online at https://www.mdpi.com/article/10.3390/ijms22105394/s1.
