# Supplementary material for: CRL4-DCAF12 Ubiquitin Ligase Controls MOV10 RNA Helicase during Spermatogenesis and T Cell Activation
Source: Int J Mol Sci. 2021 May 20;22(10):5394. doi: 10.3390/ijms22105394 (PMC8161014; doi:10.3390/ijms22105394)
Supplement: Supplementary file 1 [file ijms-22-05394-s001.zip › ijms-1215751-supplementary.pdf]

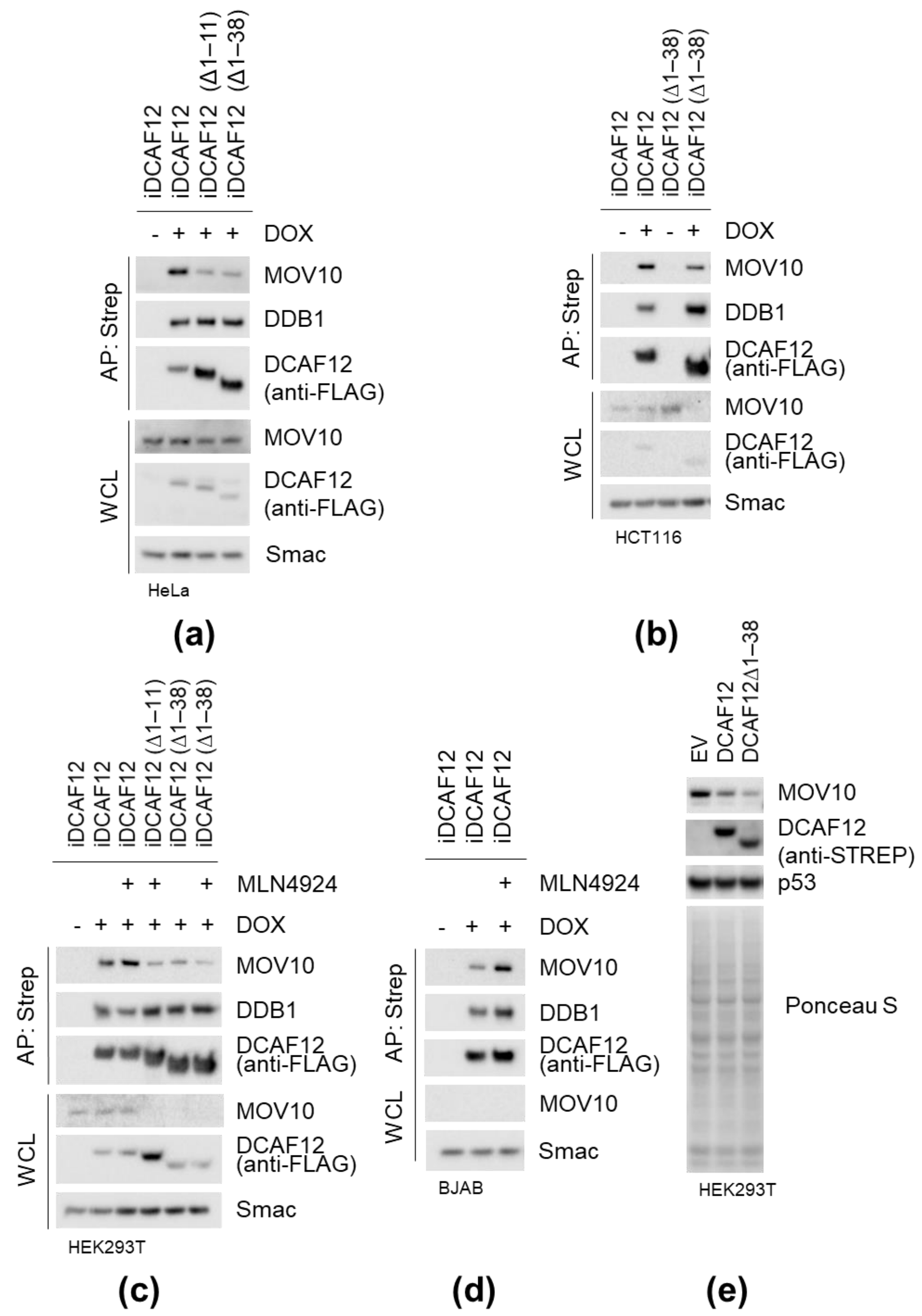

Figure S1. DCAF12 and its N-terminally truncated mutants interact with endogenous MOV10 and control MOV10 protein level in several cell lines. (**a–d**) Affinity purification (AP) of DCAF12 and its N-terminally truncated mutants from HeLa (**a**), HCT116 (**b**), HEK293T (**c**), and BJAB (**d**) stable cell lines expressing StrepII-FLAG-tagged DCAF12, DCAF12 (Δ1–11), or DCAF12 (Δ1–38) under the control of the doxycycline-inducible promoter. Where indicated, cells were treated with doxycycline (DOX) for 48 hours. MLN4924 was added to cells 6 hours before harvesting (**a–b**). In the lower panel (**c–d**), only indicated cells were treated. Whole-cell lysates were subjected to immunoblotting with indicated antibodies. (**e**) Transient expression of DCAF12 and its N-terminally truncated DCAF12 mutant in HEK293T. Cells were transiently transfected with StrepII-FLAG-tagged DCAF12 (WT) and DCAF12 (Δ1–38) and harvested after 48 hours. Whole-cell lysates were subjected to immunoblotting with indicated antibodies

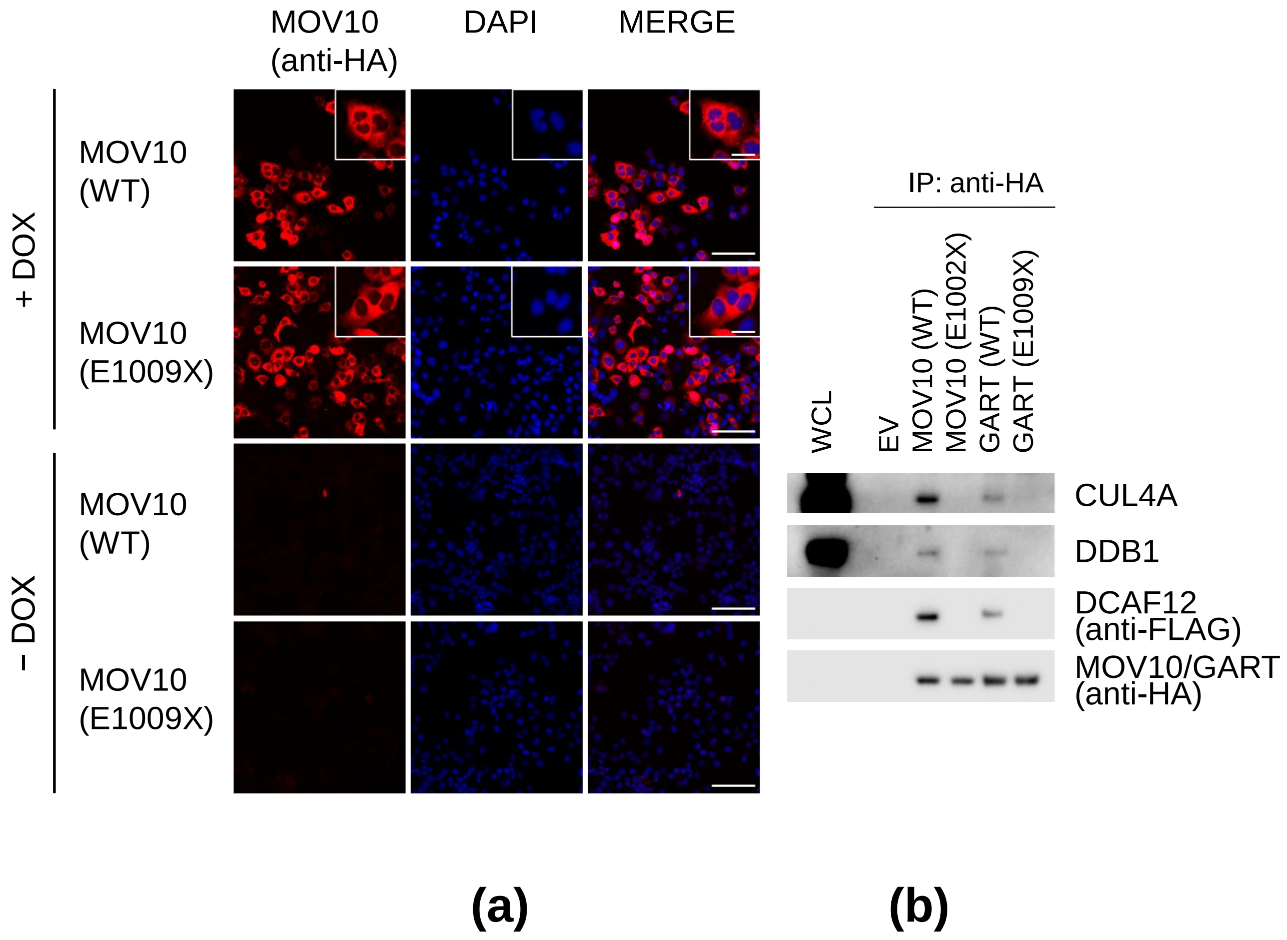

Figure S2. C-terminal -EE and -EL degrons are necessary for interaction with DCAF12 and have no apparent effect on the intracellular localisation of substrates. (**a**) Localisation of MOV10 (WT) and MOV10 (E1002X) mutants. HCT116 stable cell lines expressing HA-tagged MOV10 (WT) and MOV10 (E1002X) under the control of doxycycline-inducible promoter were treated with doxycycline (DOX) for 24 hours, fixed, and stained with anti-HA antibody to detect MOV10. DAPI was used to counterstain DNA. Scale bar, 100 µm. (**b**) Immunoprecipitation (IP) of HA-tagged MOV10 (WT), GART (WT) and their truncated mutants. HEK293T cells were co-transfected with FLAG-Twin-Strep-tagged DCAF12 and either an empty vector (EV) or HA-tagged MOV10 (WT), MOV10 (E1002X), GART (WT), and GART (E1009X). MLN4924 was added for the last 6 hours before harvesting. After lysis, whole-cell lysates (WCL) were subjected to anti-HA IP and immunoblotted as indicated.

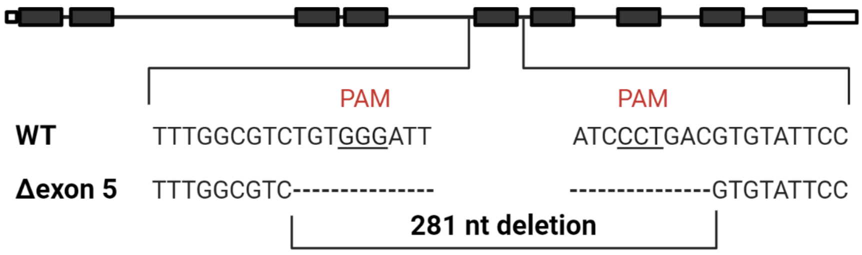

(a)

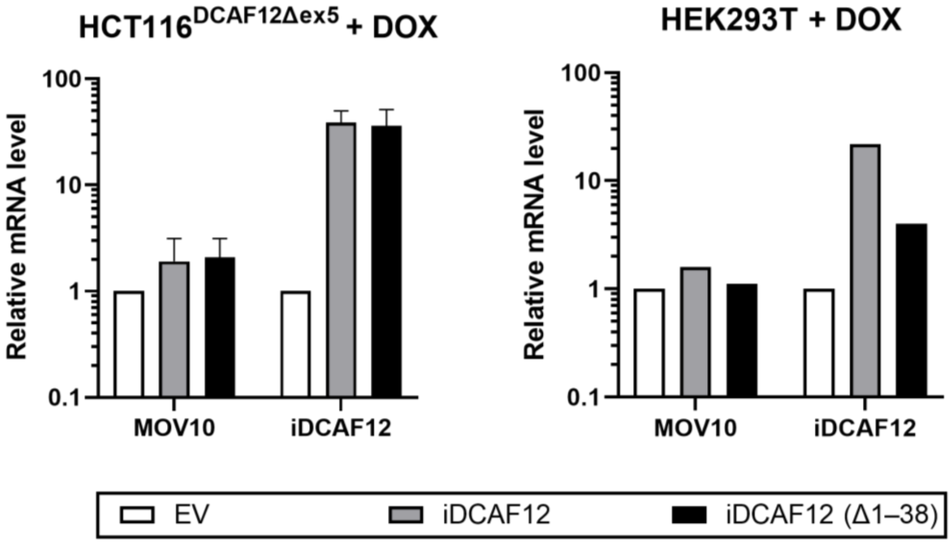

(b)

Figure S3. Generation of *DCAF12* knockout cell lines and *MOV10* relative expression after DCAF12 induction. (**a**) Schematic diagram of targeted disruption of the *DCAF12* gene using a CRISPR/Cas9 genome-editing system in HCT116 cells. Two sgRNAs were designed to target introns flanking the exon 5 of the *DCAF12* gene. PAM motives are underlined. The clone used in this study had a 281-nt deletion encompassing the exon 5. (**b**) Relative expression levels of *MOV10* mRNA in HCT116 (*DCAF12* Δexon 5) and HEK293T stable cell lines expressing StrepII-FLAG-tagged DCAF12 (WT) or DCAF12 (Δ1–38) under the control of doxycycline-inducible promoter (iDCAF12). Cells were treated with doxycycline (DOX) for 48 hours, and RT-qPCR determined relative mRNA levels of *MOV10* and *iDCAF12*. Values are presented as means ± standard deviations.

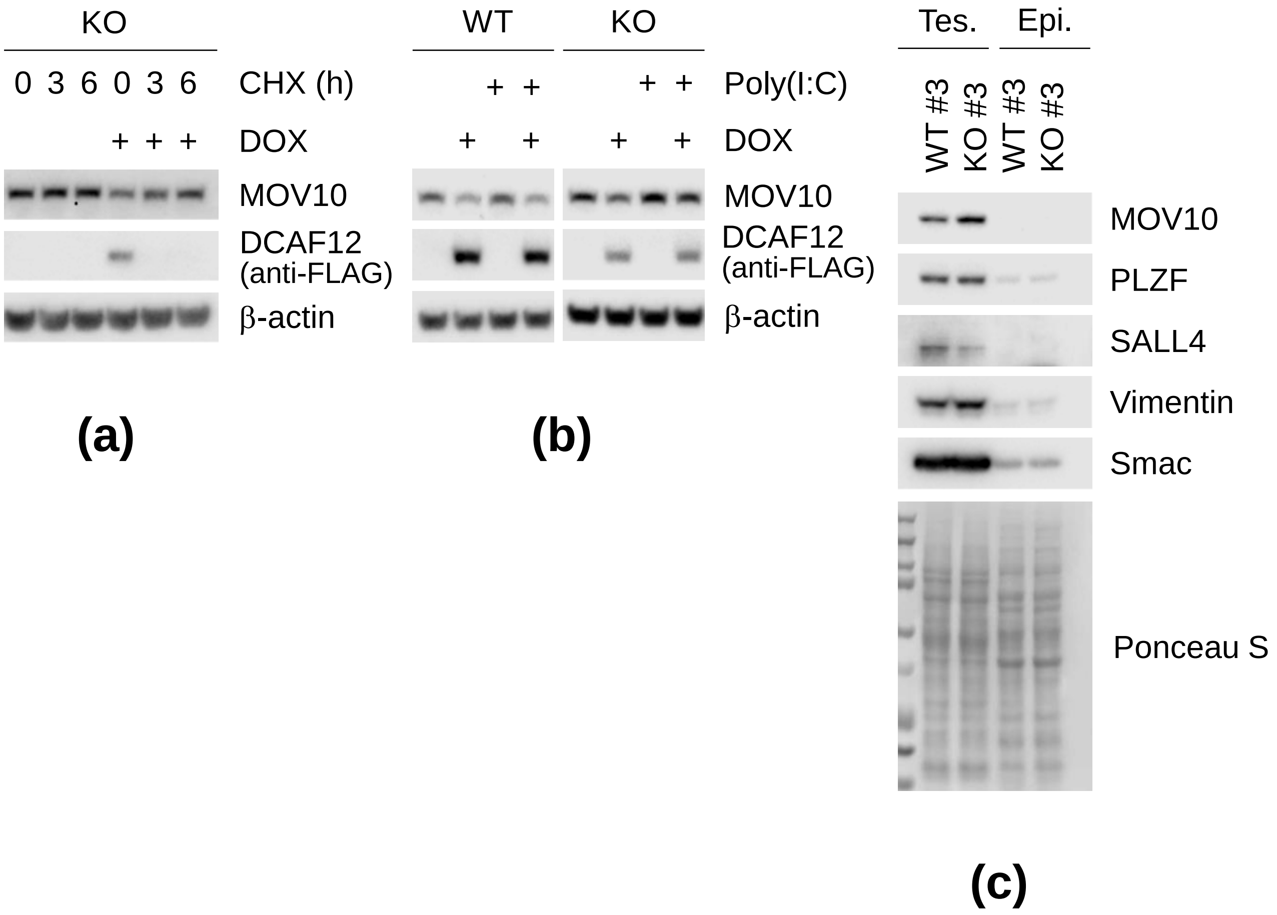

Figure S4. DCAF12 controls MOV10 protein level in mouse embryonic fibroblast (MEFs) and testes. (**a–b**) MEF whole-cell lysates immunoblotted with indicated antibodies. MEFs of indicated genotype (*Dcaf12* WT or KO) were immortalised by CRISPR/Cas9-mediated inactivation of p19ARF (**a**) or by SV40 large T antigen (**b**). Subsequently, stable cell lines expressing StrepII-FLAG-tagged DCAF12 under the doxycycline-inducible promoter control were established (**a–b**). Where indicated, cells were treated with doxycycline (DOX) for 48 hours, poly(I:C) for 24 hours, or cycloheximide (CHX) for the indicated time. (**c**) Testis (Tes.) and epididymis (Epi.) whole tissue lysates from 16-week-old *Dcaf12* WT and KO mice. Testes without the tunica albuginea and epididymis were lysed and subjected to immunoblotting with indicated antibodies

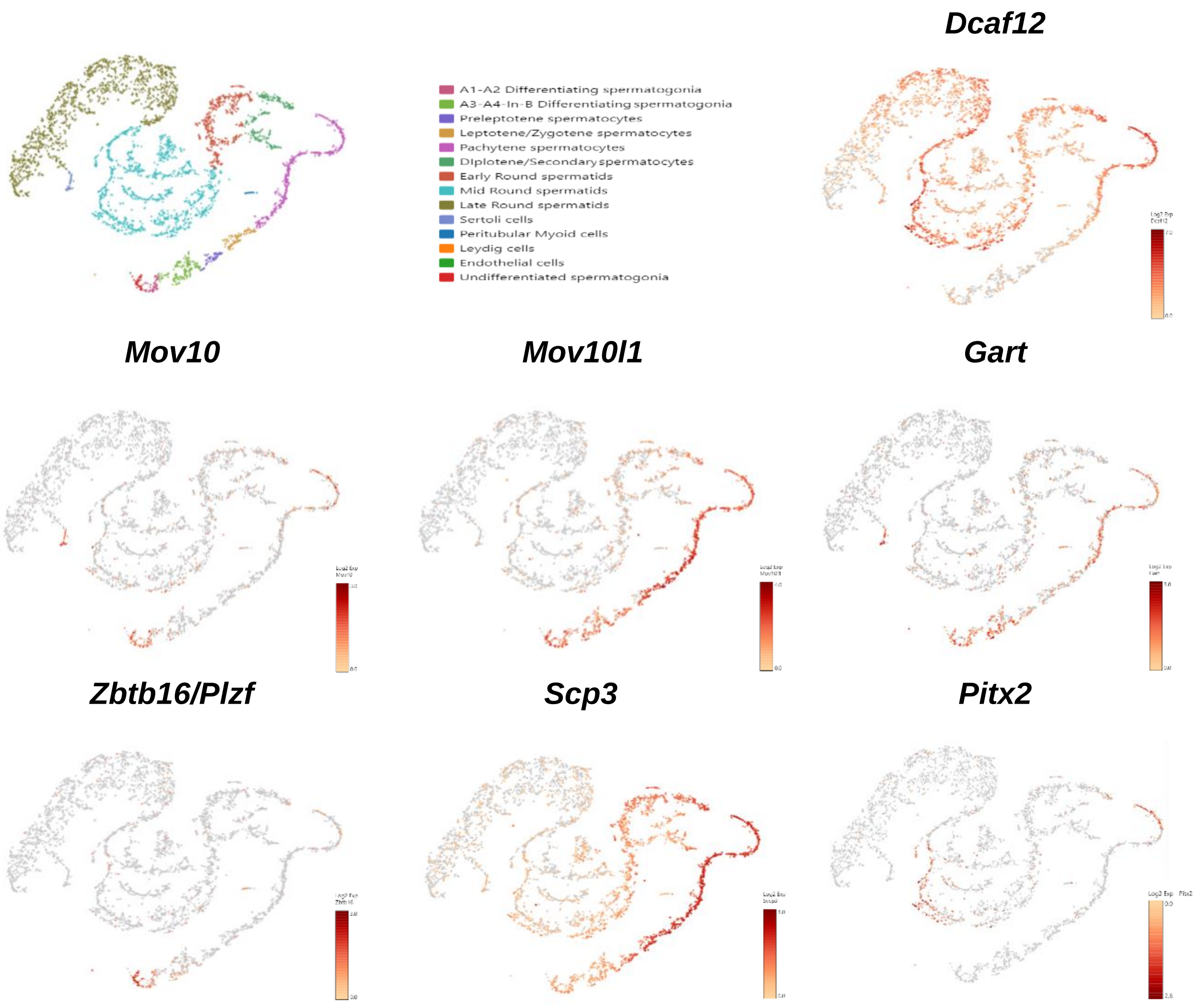

Figure S5. *Dcaf12* and *Mov10* expression during spermatogenesis. t-distributed stochastic neighbour embedding (t-SNE) plots of spermatogenic cells from publicly available spermatogenesis single-cell transcriptome datasets (doi.org/10.17632/kxd5f8vpt4.1) [1]. Colours distinguish unbiased cell clusters according to the key. Expression of *Dcaf12*, *Mov10*, *Mov10l1*, *Gart1* and indicated cell type markers used in this study is depicted.

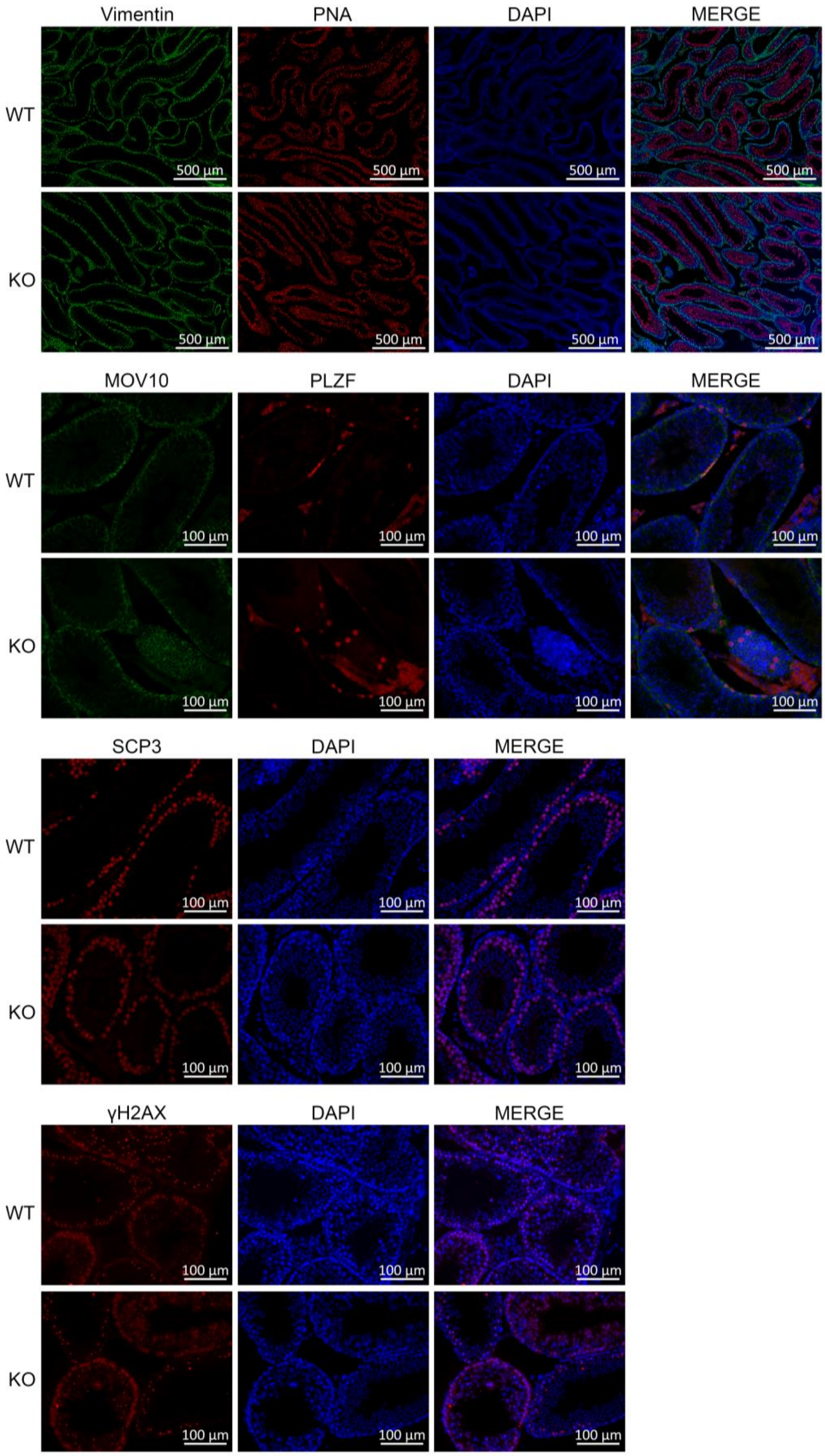

Figure S6. No apparent morphological abnormalities in *Dcaf12* KO testes.
(**a**) Immunohistological analysis of *Dcaf12* WT and KO testes. Testes from 5- to 7-week-old animals were sectioned and stained with anti-Vimentin, anti-MOV10, anti-PLZF, anti-SCP3, anti-γ-H2AX antibodies, or Alexa Fluor 594-conjugated PNA. DAPI was used to counterstain nuclei. Scale bars are as indicated. Figure continued on next page.

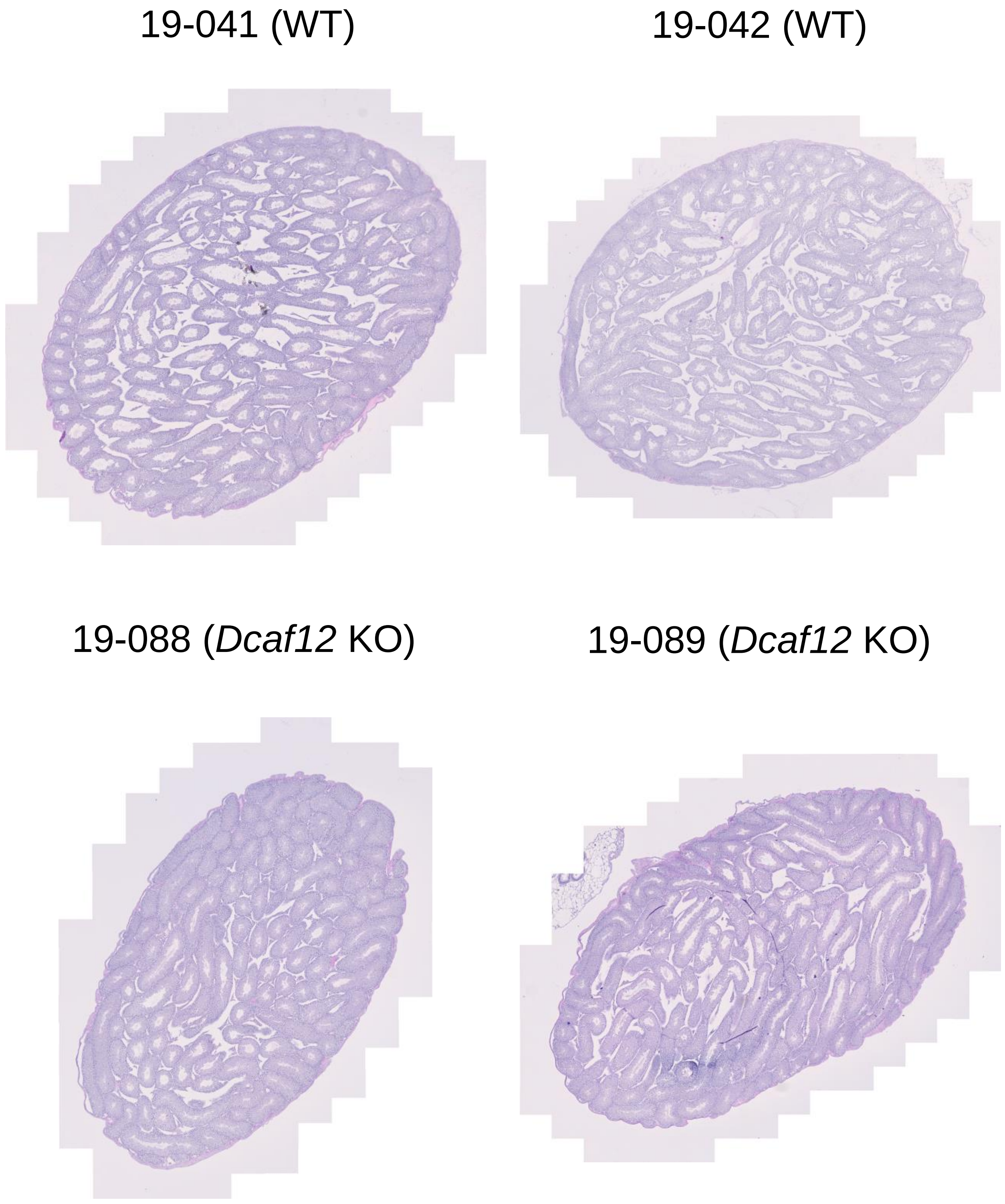

Figure S6. (continued). (**b**) A preview of high-resolution cross-sections of *Dcaf12* WT and KO testes. Testes from 5- to 7-week-old animals were fixed for 24 h in a modified Davidson's fluid and processed by standard histological methods using an automated tissue processor (Leica). Five µm-thick sections were stained with hematoxylin-eosin (Ventana Symphony H&E Slide Stainer and scanned using Axio Scan.Z1 slide scanner. Raw data are available in the Mendeley data repository (http://dx.doi.org/10.17632/h4g7ctf2wc.1).

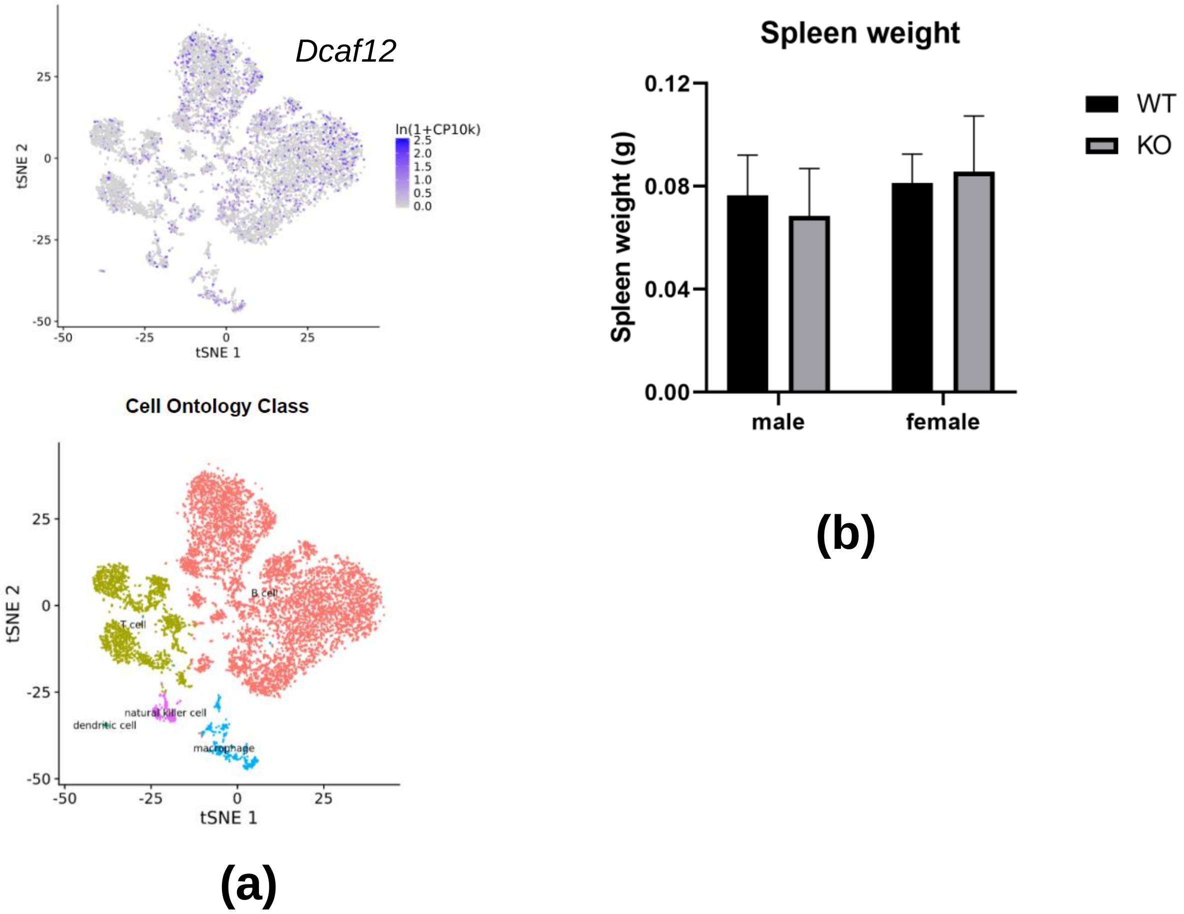

Figure S7. *Dcaf12* expression in the spleen and no difference in the spleen weight between *Dcaf12* WT and KO animals. (**a**) *Dcaf12* expression projected on publicly available t-distributed stochastic neighbour embedding (t-SNE) plot of all splenic cells prepared by microfluidic droplets [2]. Cell ontology classes are distinguished by colour according to the key. (**b**) Spleen weights of 16-week-old Dcaf12 WT and KO animals of both sexes. Data are means ± standard deviations (n = 7 per group).

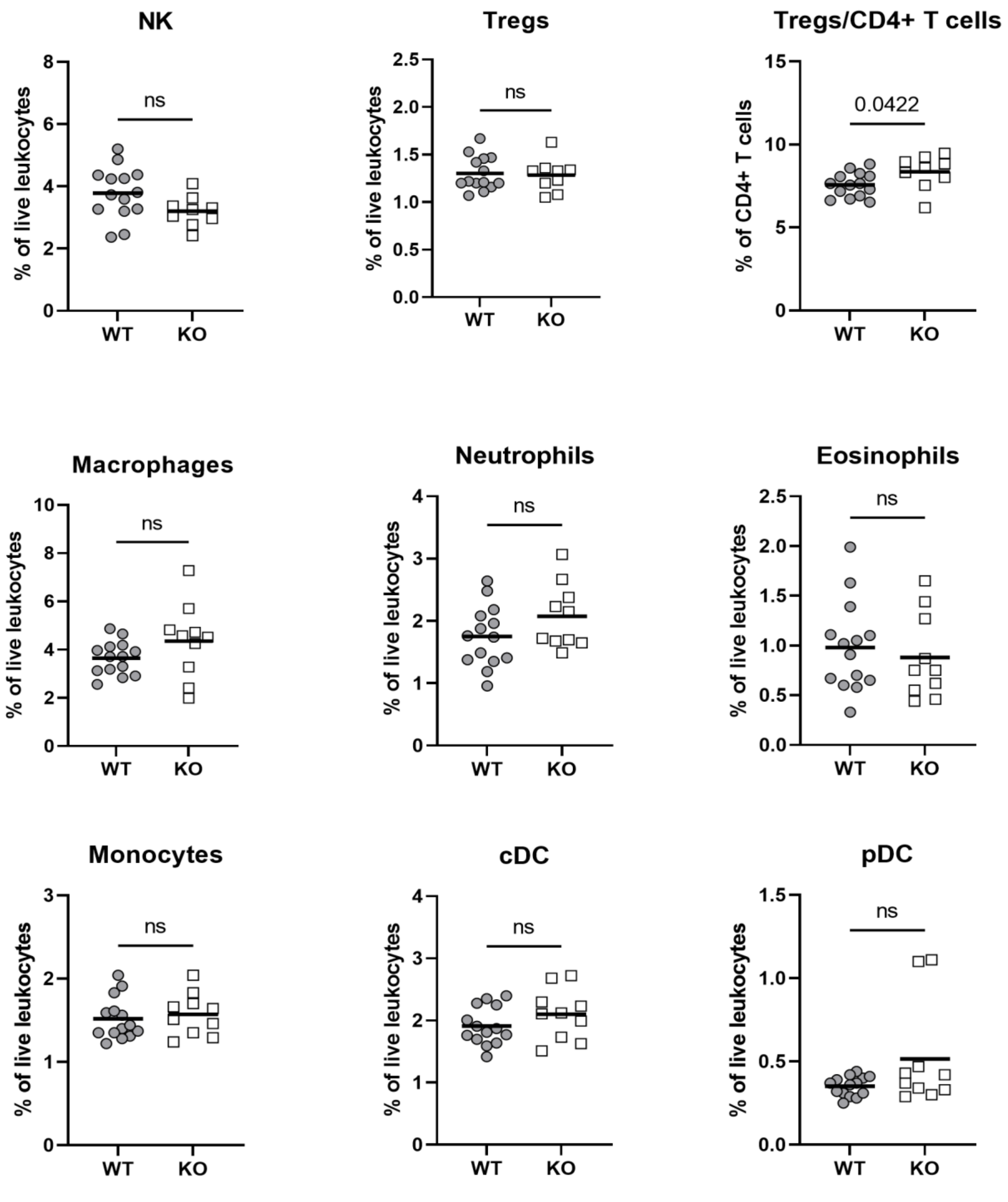

Figure S8. *Dcaf12* deficiency leads to dysregulation of immune cell populations (related to Figure 8). Quantification of immune cell populations in the spleen of 16- to 20-week-old Dcaf12 WT and KO female mice. Immune cell populations were analysed by flow cytometry. Individual data points and means are shown (n = 10–14 per group). Statistical significance was assessed by an unpaired two-tailed t-test. P-value < 0.05 was considered significant; ns = not significant

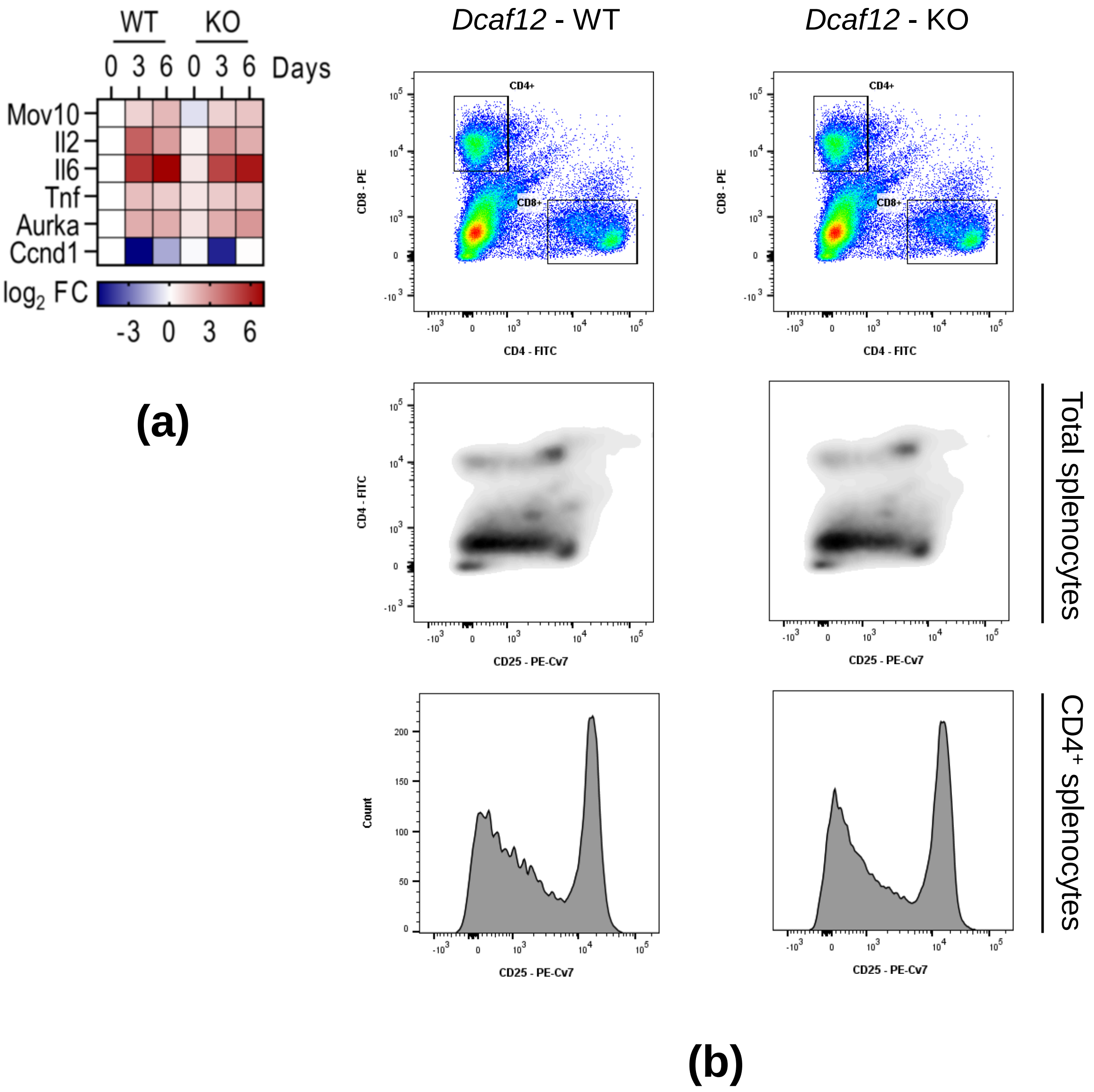

Figure S9. No apparent differences in activation of Dcaf12-deficient T cells (related to Figure 9b). (**a**) Relative mRNA expression levels of indicated genes in CD3/CD28-activated splenocytes from Figure 7b were determined by RT-qPCR. The heat map shows log_2 fold change (FC) of gene expression relative to the non-activated state of Dcaf12 WT splenocytes. (**b**) Flow cytometry analysis of splenocytes from Figure 7b. Following antibodies were used: Fc block (1:200; 2G4; BD Biosciences), CD4-FITC (1:600; RM4-5; BD Biosciences), CD8a-PE-CF594 (1:300; 53-6.7; BD Biosciences), CD25-PE-CY7 (PC61; BD Biosciences).

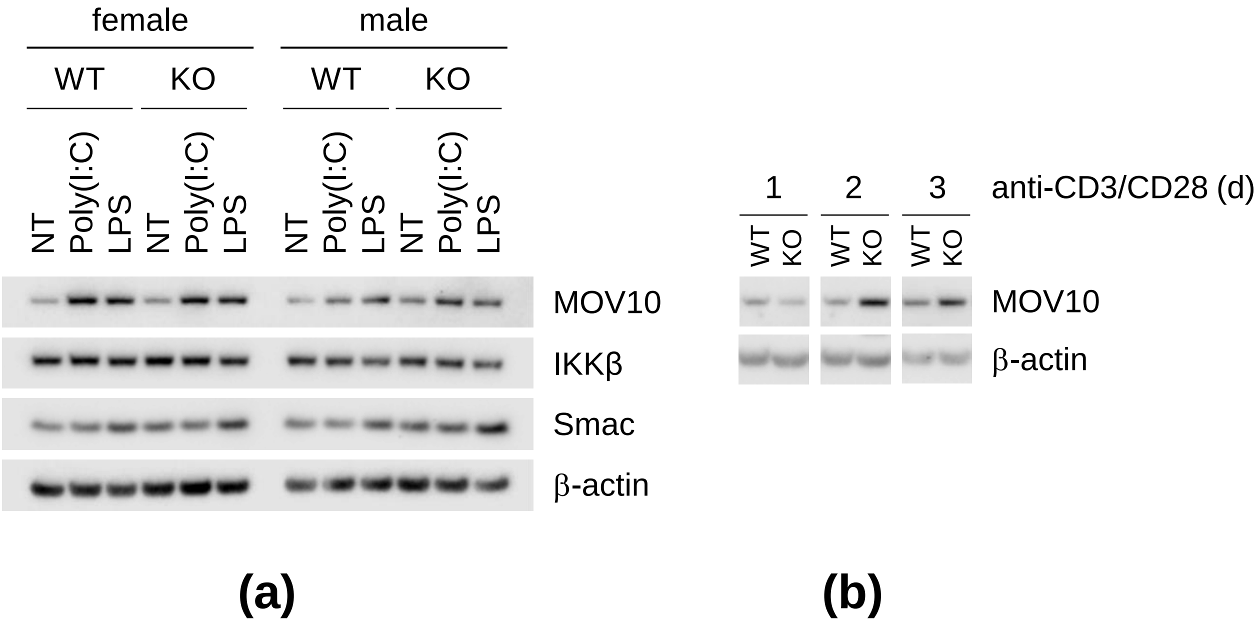

Figure S10. MOV10 is upregulated in splenocytes treated with various stimuli. (**a**) Splenocyte whole-cell lysates obtained from poly(I:C)- and LPS-stimulated splenocytes. Freshly isolated splenocytes from *Dcaf12* WT and KO mice of indicated sexes were stimulated with poly(I:C) and LPS for 24 hours, lysed, and immunoblotted with indicated antibodies. (**b**) Soluble fractions of lysates obtained from CD3/CD28-activated splenocytes. Splenocytes isolated from *Dcaf12* WT and KO mice were activated with Dynabeads (anti-CD3/CD28) for indicated times, lysed, and the soluble fractions were immunoblotted with indicated antibodies.

Table S1. List of primers used in this study

Primer name	Sequence	Used for
MOV10_f_BamHI	GGGGGATCCCCCAGTAAGTTCAGCTGCCG	Cloning into pcDNA3.1-HA
MOV10_r_XhoI	GGGCTCGAGTCAGAGCTCATTCCTCCACTCT	
MOV10_E1002X_r_XhoI	GGCTCGAGTCAATTCCTCCACTCTGGCTCCACTTG	
GART_f_BclI	GGGTGATCAGCAGCCCGAGTACTTATAATTGG	
GART_r_XhoI	GGGCTCGAGTCATTCCTCTTTAACCCAACAGATCT	
GART_E1009X_r_XhoI	GGGCTCGAGTCATTTAACCCAACAGATCTTGCCATT	
CUL4A_f_BamHI	GGGGGATCCGCGGACGAGGCCCCGCGGAAGGGCAGCTTCT	
CUL4A_r_NotI	GGGGCGGCCGCTCAGGCCACGTAGTGGTACTGA	
DCAF12_f_XbaI	GGGTCTAGAGCCCGGAAAGTAGTTAGCAGGAA	Cloning into pcDNA3.1-NSF
DCAF12_r_SalI	GGGGTCGACTTAACTCCAGAGCCCAGCATAG	
DCAF12L_f_NheI	GGGGCTAGCGCCCAGCAGCAAACAGGTAG	
DCAF12L1_r_SalI	GGGGTCGACTTAGCTCCAGAGGCCTGCAT	
DCAF12L2_r_SalI	GGGGTCGACTTAACTCCAGAGGCCTGCGT	
DCAF12L2new_r_SalI	GGGGTCGACACAAATGCAGCATGAAGAAGGAA	
DCAF12_Δ1-11_f_SpeI	GGGACTAGTGCGCCCGCCTCGCCGGGAGC	
DCAF12_Δ1-38_f_SpeI	GGGACTAGTCTTCCTCCTGTGAAGAGATC	
NSF_f_AgeI	GGGACCGGTGCCACCATGGATTATAAAGAT	Subcloning into pTRIPZ
NSF_r_XhoI_SalI	GGGTCGACTCGAGAGGTGACACTATAGAATAGGGCCC	
NSF_f_SfiI	AAAGGCCTCTGAGGCCACCATGGATTATAAAGATGATGATGAT AAAG	Subcloning into pSBtet-Pur
NSF_r_SfiI	CTTGGCCTGACAGGCCATAGGGCCCTCTAGATGCATGCTCG	
HA_f_SfiI	AAAGGCCTCTGAGGCCACCATGGCGTACCCCTACGACGTGCC CGACTA	
D12_F1	CACCGAAGCCCTCTTTGGCGTCTGT	Oligo pairs ligated into pXPR001
D12_F1_R	AAACACAGACGCCAAAGAGGGCTTC	
D12_R1	CACCGAAGCAAGAGGAATACACGTC	
D12_R1_R	AAACGACGTGTATTCCTCTTGCTTC	
pXPR001-test_F	TTGCATATACGATACAAGGCTGTT	Verification of insertion
pXPR001-test_R	CGGTGCCACTTTTTCAAGTT	
hDCAF12_f	GAGGATGATGCACAAACAGC	Verification of exon 5 deletion
hDCAF12_r	CCAGAATACAGCACCTGCAA	
mDcaf12_f	ACTGCCAGGCGTATTGTACC	Verification of exon 4 deletion
mDcaf12_r	GCACAGGCAGGTGGATCTAT	

Primers for quantitative Real-Time PCR

Primer name	Forward	Reverse
MOV10	CTGCAAGGTCTGAGCAAGC	GAGGTAGTCATGGCTGTGGG
DCAF12	TGCAAAGTGATCCAGCCCTA	GTGACACCAACTTCCCACAC
iDCAF12	CCCCAATGCTGTTTACACCC	AATCCTCTCCGCTAGCTTAACTCCAGAGCCCAGCATAG
RPS13	CAGTCGGCTTTACCCTATCG	CCCTTCTTGGCCAGTTTGTA
Mov10	GTCCTCCGGGCTGTTTAGG	CGCGGAAATGAAACCCGAG
Il2	GAAACTCCCCAGGATGCTCA	CGCAGAGGTCCAAGTTCATCT
Il6	CTGCAAGAGACTTCCATCCAGTT	GAAGTAGGGAAGGCCGTGG
Tnf	CATCTTCTCAAAATTCGAGTGACAA	TGGGAGTAGACAAGGTACAACCC
Aurka	CAGAGAACAGCTACTTACATC	GTCTGCAATCTTCAACTCTC
Ccnd1	AACACTTCCTCTCCAAAATG	GAACTTCACATCTGTGGC
Rps13	ACTCCCTCCCAGATAGGTGTAA	AGTCACAAAACGGACCTGGG

Table S2. List of antibodies used in this study

Antigen	Source/clonality	Obtained from	Cat. #
ADAR1	rabbit/poly	antibodies-online	ABIN2855100
Cleaved caspase-3 (Asp175)	rabbit/poly	Cell Signaling	9661
Cleaved PARP (Asp214)	rabbit/poly	Cell Signaling	9544
CUL-4	mouse/mono	Santa Cruz	sc-377188
CUL-4A	rabbit/poly	Abcam	ab72548
DCAF7	rabbit/poly	Novus Biologicals	NBP1-92589
DDB1	rabbit/poly	Invitrogen	34-2300
DDB1	mouse/mono	Santa Cruz	sc-376860
FBXO28	rabbit/poly	Bethyl	A302-377A
FLAG	rabbit/mono	Cell Signaling	14793
FLAG M2	mouse/mono	Sigma	F1804
GART	mouse/mono	Santa Cruz	sc-73408
HA	rabbit/mono	Cell Signaling	3724
Histone γH2AX	mouse/mono	Millipore	05-636
IKKβ	mouse/mono	Cell Signaling	8943
MOV10	mouse/mono	Santa Cruz	sc-515722
MOV10	rabbit/poly	Bethyl	A301-571A-T
p21	rabbit/poly	Santa Cruz	sc-397
p27	mouse/mono	BD Transduction L.	610242
p53	rabbit/mono	Cell Signaling	2527
Phospho-β-catenin (Ser33/37)	rabbit/poly	Cell Signaling	2009
PITX2	mouse/mono	Santa Cruz	sc-390457
SALL4	mouse/mono	Santa Cruz	sc-101147
SCP3	mouse/mono	Santa Cruz	sc-74569
SKP1	rabbit/poly	Cell Signaling	2156
Smac/Diablo	rabbit/mono	Cell Signaling	15108
Strep II Tag	mouse/mono	Novus Biologicals	NBP2-43735
Vimentin (D21H3) XP	rabbit/mono	Cell Signaling	5741
WDR33	mouse/mono	Santa Cruz	sc-374466
WDR5	mouse/mono	Santa Cruz	sc-393080
WDR74	mouse/mono	Santa Cruz	sc-393822
XIAP	mouse/mono	Santa Cruz	sc-55551
ZBTB16 (PLZF)	rabbit/poly	Atlas Antibodies	HPA001499
α-tubulin	mouse/mono	Proteintech	66031-1-Ig
β-actin	mouse/mono	Santa Cruz	sc-69879

Supplementary methods

1. Messenger RNA quantification

Total RNA was purified from cells using the RNeasy Mini Kit (QIAGEN). 1000 ng of total RNA was reverse-transcribed using Maxima H Minus cDNA SynthesisMaster Mix (Thermo Fisher Scientific), and obtained cDNA was used as a template for qPCR. The reaction was performed using the LightCycler 480 SYBR Green I Master Mix and the LightCycler 480 instrument (Roche). *RPS13* was used for normalisation. Primer sequences used for RT-qPCR are listed in Table S1.

2. Immunohistochemistry

Mouse testes were fixed in modified Davidson's fluid and processed as described elsewhere [3] before being embedded in paraffin. Testis sections (4-6 μm thick) were deparaffinised, hydrated, and subjected to antigen retrieval in 0.01 M citrate buffer (pH 6) in a steam bath for 18 minutes. Next, they were permeabilised with 0.2% Triton X-100 in PBS for 10 minutes, blocked with 5% goat and 5% horse serum for 1 hour, and incubated overnight at 4 ° C with the following primary antibodies: anti-Vimentin (Cell Signaling; 5741; dilution 1:400), anti-MOV10 (Santa Cruz; sc-515722; dilution 1:100), anti-PLZF (Atlas Antibodies; HPA001499; dilution 1:200), anti-SCP3 (Santa Cruz; sc-74569; dilution 1:400), anti-γH2AX (Millipore; 05-636; dilution 1:1000). Alexa Fluor 555- and Alexa Fluor 647-conjugated anti-rabbit/mouse IgGs were used as secondary antibodies. Alexa Fluor 594-conjugated PNA was used to visualise acrosomes of spermatids, and nuclei were counterstained with DAPI. Slides were mounted with ProLong Gold Antifade Mountant (Thermo Fisher Scientific), and image acquisition was performed using a Zeiss Axio Imager.Z2 microscope, equipped with Zeiss ZEN software.

3. Mass spectrometry analysis of DCAF12-associated proteins

Protein Digestion

After acetone precipitation, proteins were resuspended in 100 mM TEAB containing 1% SDC. Cysteines were reduced with 5 mM TCEP (60 ° C for 60 minutes) and blocked by MMTS (RT for 10 minutes). Proteins were "on-bead" digested with trypsin (1 μg) at 37 ° C overnight. After digestion, samples were centrifuged, and supernatants were collected and acidified with 1% TFA. SDC was removed by extraction with ethyl acetate [4]. Peptides were desalted using in-house made stage tips packed with C18 disks (Empore), according to Rappsilber et al. [5].

nLC-MS 2 Analysis

Nano Reversed-phase column (EASY-Spray column, 50 cm x 75 μm ID, PepMap C18, 2 μm particles, 100 Å pore size) was used for LC/MS analysis. Mobile phase buffer A was composed of water and 0.1% formic acid. Mobile phase B was composed of acetonitrile and 0.1% formic acid. Samples were loaded onto the trap column (Acclaim PepMap300, C18, 5 μm, 300 Å Wide Pore, 300 μm x 5 mm, 5 Cartridges) for 4 minutes at 15 μl/min. Loading buffer was composed of water, 2% acetonitrile and 0.1% trifluoroacetic acid. Peptides were eluted with a mobile phase B gradient from 4% to 35% B during 60 minutes. Eluting peptide cations were converted to gas-phase ions by electrospray ionization and analysed on a Thermo Orbitrap Fusion (Q-OT- qIT, Thermo). Survey scans of peptide precursors from 400 to 1600 m/z were performed at 120K resolution (at 200 m/z) with a 5×10^5 ion count targets. Tandem MS was performed by isolation at 1.5 Th with the quadrupole, HCD fragmentation with a normalised collision energy of 30, and rapid scan MS analysis in the ion trap. The MS 2 ion count target was set to 10^4 , and the max injection time was 35 milliseconds. Only those precursors with charge state 2–6 were sampled for MS 2. The dynamic exclusion duration was set to 45 seconds with a 10ppm tolerance around the selected precursor and its isotopes. Monoisotopic precursor selection was turned on. The instrument was run in the top speed mode with 2 second cycles [6].

Data Analysis

All data were analysed and quantified with the MaxQuant software (version 1.5.3.8) [7]. The false discovery rate (FDR) was set to 1% for both proteins and peptides, and we specified a minimum length of seven amino acids. The Andromeda search engine was used for the MS/MS spectra search against a human UniProt database. Enzyme specificity was set as C-terminal to Arg and Lys, allowing cleavage at proline bonds and a maximum of two missed cleavages. Dithiomethylation of cysteine was selected as fixed modification and N-terminal protein acetylation, methionine oxidation and serine/threonine/tyrosine phosphorylation as variable modifications. The "match between runs" feature of MaxQuant was used to transfer identifications to other LC-MS/MS runs based on their masses and retention time (maximum deviation 0.7 min), and this was also used in quantification experiments. Quantifications were performed with the label-free algorithms [8]. Data analysis was performed using Perseus 1.5.2.4 software.

Supplementary references

1. Hermann, B.P.; Cheng, K.; Singh, A.; Roa-De La Cruz, L.; Mutoji, K.N.; Chen, I.C.; Gildersleeve, H.; Lehle, J.D.; Mayo, M.; Westernströer, B.; et al. The Mammalian Spermatogenesis Single-Cell Transcriptome, from Spermatogonial Stem Cells to Spermatids. *Cell Rep.* **2018**, *25*, 1650-1667.e8, doi:10.1016/j.celrep.2018.10.026.
2. Tabula, T.; Consortium, M. Single-cell transcriptomics of 20 mouse organs creates a Tabula Muris. *Nature* **2018**, doi:10.1038/s41586-018-0590-4.
3. Latendresse, J.R.; Warbrittion, A.R.; Jonassen, H.; Creasy, D.M. Fixation of testes and eyes using a modified Davidson's fluid: Comparison with Bouin's fluid and conventional Davidson's fluid. *Toxicol. Pathol.* **2002**, *30*, 524–533, doi:10.1080/01926230290105721.
4. Masuda, T.; Tomita, M.; Ishihama, Y. Phase transfer surfactant-aided trypsin digestion for membrane proteome analysis. *J. Proteome Res.* **2008**, *7*, 731–740, doi:10.1021/pr700658q.
5. Rappsilber, J.; Mann, M.; Ishihama, Y. Protocol for micro-purification, enrichment, pre-fractionation and storage of peptides for proteomics using StageTips. *Nat. Protoc.* **2007**, *2*, 1896–1906, doi:10.1038/nprot.2007.261.
6. Hebert, A.S.; Richards, A.L.; Bailey, D.J.; Ulbrich, A.; Coughlin, E.E.; Westphall, M.S.; Coon, J.J. The one hour yeast proteome. *Mol. Cell. Proteomics* **2014**, *13*, 339–347, doi:10.1074/mcp.M113.034769.
7. Cox, J.; Hein, M.Y.; Luber, C.A.; Paron, I.; Nagaraj, N.; Mann, M. Accurate proteome-wide label-free quantification by delayed normalization and maximal peptide ratio extraction, termed MaxLFQ. *Mol. Cell. Proteomics* **2014**, *13*, 2513–2526, doi:10.1074/mcp.M113.031591.
8. Tyanova, S.; Temu, T.; Sinitcyn, P.; Carlson, A.; Hein, M.Y.; Geiger, T.; Mann, M.; Cox, J. The Perseus computational platform for comprehensive analysis of (prote)omics data. *Nat. Methods* **2016**, *13*, 731–740, doi:10.1038/nmeth.3901.
